# TAK1 modulates satellite stem cell homeostasis and skeletal muscle repair

**DOI:** 10.1038/ncomms10123

**Published:** 2015-12-09

**Authors:** Yuji Ogura, Sajedah M. Hindi, Shuichi Sato, Guangyan Xiong, Shizuo Akira, Ashok Kumar

**Affiliations:** 1 Department of Anatomical Sciences and Neurobiology, University of Louisville School of Medicine, Louisville, Kentucky 40202, USA; 2 Laboratory of Host Defense, WPI Immunology Frontier Research Center, Osaka University, Osaka 565-0871, Japan

## Abstract

Satellite cells are resident adult stem cells that are required for regeneration of skeletal muscle. However, signalling mechanisms that regulate satellite cell function are less understood. Here we demonstrate that transforming growth factor-β-activated kinase 1 (TAK1) is important in satellite stem cell homeostasis and function. Inactivation of TAK1 in satellite cells inhibits muscle regeneration in adult mice. TAK1 is essential for satellite cell proliferation and its inactivation causes precocious differentiation. Moreover, TAK1-deficient satellite cells exhibit increased oxidative stress and undergo spontaneous cell death, primarily through necroptosis. TAK1 is required for the activation of NF-κB and JNK in satellite cells. Forced activation of NF-κB improves survival and proliferation of TAK1-deficient satellite cells. Furthermore, TAK1-mediated activation of JNK is essential to prevent oxidative stress and precocious differentiation of satellite cells. Collectively, our study suggests that TAK1 is required for maintaining the pool of satellite stem cells and for regenerative myogenesis.

## Abstract (web summary)

TAK1 is a MEK kinase family member that activates pro-survival NF-kB signalling but also pro-apoptotic caspase signalling in various cell types. Here the authors show that satellite-cell specific deletion of TAK1 in mice depletes the muscle stem cell pool and thus limits myofibre regeneration after injury.

## Graphical abstract

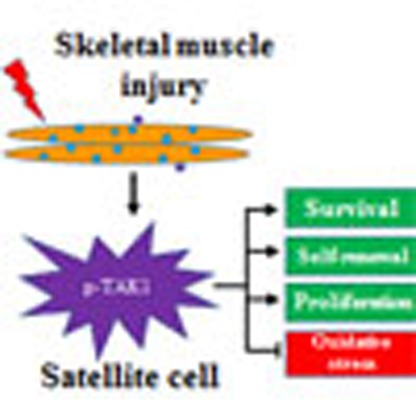

Skeletal muscle regeneration is mediated by a specialized population of adult stem cells known as satellite cells, which reside adjacent to myofibers^1^. Although normally quiescent, satellite cells are activated upon muscle damage to proliferate, differentiate and fuse to form new myofibers leading to regeneration of damaged tissue and restoration of normal function^2^,^3^. Satellite cells are inextricably linked to the paired box transcription factor Pax7 (ref. 4). Satellite cells of all mammalian species studied so far have been found to express Pax7 (ref. 1). Inactivation of Pax7 results in severe depletion of muscle stem cells in adult animals^4^. Pax7 along with myogenic regulator factor, MyoD, determines the fate of satellite cells. Although all quiescent satellite cells express Pax7, they do not express MyoD protein. Upon muscle injury, satellite cells proliferate and rapidly induce MyoD expression, leading to their progression in the myogenic lineage to generate fusion competent myoblasts^3^. Although the majority of activated satellite cells (that is, Pax7^+^/MyoD^+^) differentiate into myogenic lineage through ensuing repression of Pax7 and upregulation of other myogenic regulatory factors, such as myogenin and MRF4, a small proportion self-renew and return to the quiescent state (that is, Pax7^+^/MyoD^−^) to respond to future muscle injury and repair^2^,^5^.

Satellite cell self-renewal, proliferation and differentiation are regulated through the activation of multiple signalling pathways. Activation of Notch signalling promotes satellite cell self-renewal and inhibits differentiation through repressing the expression of MyoD^5^,^6^,^7^,^8^. Moreover, the Wnt7a/Fzd7 planar-cell-polarity pathway drives the symmetric expansion of satellite stem cells to enhance regeneration of injured myofibers^9^. MAPK signalling pathways also regulate the self-renewal and differentiation of satellite cells. Angiotensin-1 binds to Tie-2 receptor to increase the number of quiescent satellite cells through the activation of the ERK1/2 signalling pathway^10^. Moreover, c-Jun N-terminal kinase (JNK) signalling seems to promote satellite cell expansion during regenerative myogenesis^11^. By contrast, the activation of p38 MAPK inhibits self-renewal and promotes differentiation of satellite cells into myoblasts^12^,^13^,^14^. Although the canonical nuclear factor-kappa B (NF-κB) pathway activated through inhibitor of kappa B kinase-β (IKKβ) blocks terminal differentiation of myogenic cells^15^,^16^, in the context of cancer cachexia stimulation of NF-κB promotes the expansion of satellite cells^17^. Furthermore, the activation of JAK–STAT signalling negatively regulates satellite stem cell proliferation and function during regenerative myogenesis^18^,^19^.

Transforming growth factor-β-activated kinase 1 (TAK1), a member of the MEK kinase family, is an important signalling protein that activates several signalling pathways in response to cytokines, growth factors and microbial products^20^,^21^. TAK1 constitutively interacts with accessory protein TAB1 and also with TAB2 or TAB3 (refs 21, 22, 23, 24, 25). TAB1 constitutively binds and activates TAK1, whereas TAB2 or TAB3 bind TAK1 after stimulation^26^. The TAK1 complex is activated in response to proinflammatory stimuli via K63-linked polyubiquitination driven by the E2 ligase UBC13/UEV1A and the RING finger E3 ligases TRAF2 or TRAF6. K63-linked polyubiquitination at the K158 residue of TAK1 by TRAF6/UBC13/UEV1A is an important response to stimulation of cells by cytokines and microbial products^27^,^28^. TAB2 and TAB3 preferentially bind to K63-linked polyubiquitin chains with strong affinity to activate TAK1 (ref. 29), and even free, unconjugated, K63-linked polyubiquitin chains can activate TAK1 (refs 30, 31). Strong interactions between unanchored K63 polyubiquitin chains and TAB2/TAB3 change the conformation of TAK1 resulting in its autophosphorylation. TAK1 polyubiquitination induces autophosphorylation at Thr187, within its activation loop, and other sites, including Thr184 and Ser192 (refs 32, 33).

Proinflammatory and stress signals stimulate TAK1 to induce both proapoptotic and anti-apoptotic signals^22^. TAK1 mediates the pro-survival signal by inducing the nuclear translocation of NF-κB and the activation of c-Jun N-terminal kinases (JNKs), whereas the proapoptotic signal is mediated by the activation of caspases^22^,^34^. Germline deletion of TAK1 or its binding partners TAB1 or TAB2 in mice leads to embryonic lethality, suggesting that TAK1-mediated signalling is essential for embryogenesis^21^,^34^. Tissue-specific knockout mice have demonstrated that TAK1 has important functions in innate and adaptive immune responses, vasculature development, survival of keratinocytes, haematopoietic cells and hepatocytes, and morphogenesis, growth and maintenance of cartilage^21^,^35^,^36^,^37^. Although apoptosis is the major mode of cell death in physiological conditions, necroptosis, which involves the formation of a receptor-interacting protein (RIP)1 and RIP3 complex (called necrosome), is critical in many pathological conditions^38^,^39^. Depending on upstream stimuli, TAK1 can promote or inhibit necroptosis in different cell types^34^,^40^,^41^,^42^.

TAK1 is highly expressed and activated in developing skeletal muscle of mice. We and others have previously reported that TAK1 promotes differentiation of C2C12 myoblasts into myotubes through the activation of p38 MAPK^43^,^44^. Although TAK1 seems essential for myogenesis, the role of TAK1 in satellite stem cell homeostasis and function has not been investigated. Through the generation of inducible satellite cell-specific TAK1 knockout mice, here we investigate the role and mechanisms of action of TAK1 in satellite cell survival, proliferation and function during regenerative myogenesis. Our results demonstrate that TAK1 is essential for the survival and proliferation of satellite cells both *in vivo* and *in vitro*. Inactivation of TAK1 in satellite cells induces oxidative stress and causes spontaneous cell death, primarily through necroptosis. TAK1 is required for the activation of JNKs and NF-κB in satellite cells. Forced expression of a constitutively active mutant of IKKβ improves proliferation and survival of TAK1-deficient satellite cells. Moreover, overexpression of JNK1 reduces oxidative stress and augments the expression of Pax7 in TAK1-deficient satellite cells.

## Results

### TAK1 is required for the regeneration of myofibers upon injury

**Figure 1:**
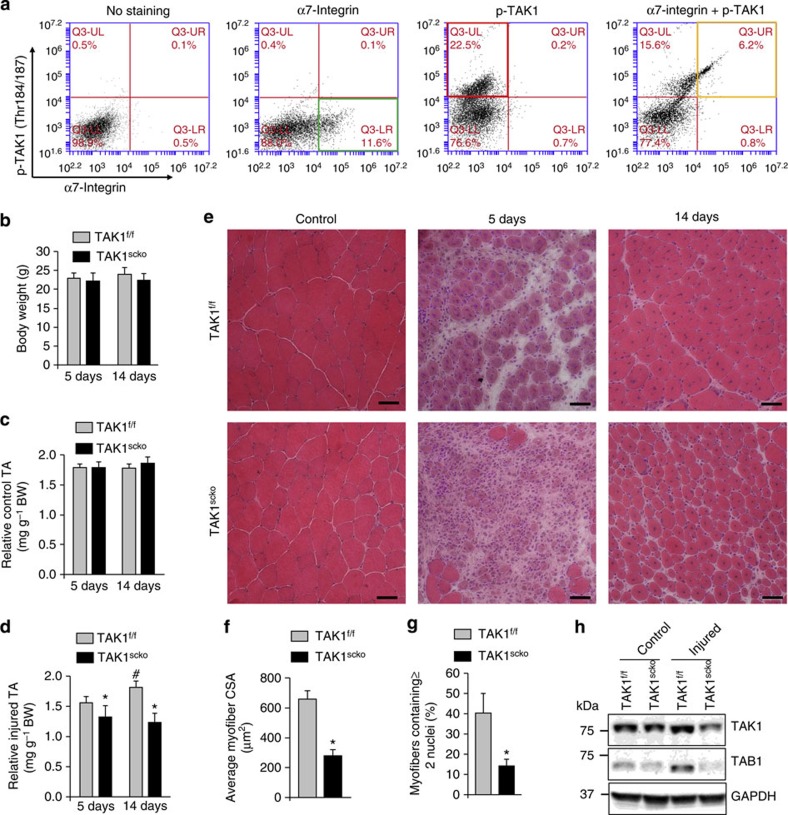
TAK1 in satellite cells is required for regeneration of muscle fibres upon injury. (**a**) Primary mononucleated cells were isolated from hind limb muscle of WT mice and subjected for FACS analysis for the expression of α7-integrin and phospho-TAK1 (Thr184/187) immediately after isolation. Representative dot plots presented here demonstrate enrichment of phospho-TAK1^+^ cells amongst α7-intigrin^+^ population. *N*=3 in each group. (**b**) Following tamoxifen-mediated Cre recombination, tibial anterior (TA) muscle of adult TAK1^f/f^ and TAK1^scko^ mice were injected with 100 μl of 1.2% BaCl_2_ solution after which muscles were isolated and analysed at day 5 and 14. Relative (**b**) absolute body weight; (**c**) uninjured TA muscle weight; and (**d**) injured TA muscle weight of TAK1^f/f^ and TAK1^scko^ mice 5d after injury. *N*=6 in each group. (**e**) Representative photomicrographs of H&E-stained sections illustrating a severe regeneration defect in injured TA muscle of TAK1^scko^ mice compared with TAK1^f/f^ littermates at day 5 (*N*=6) and 14 (*N*=3) after BaCl_2_-mediated injury. Scale bar, 50 μM. Quantification of (**f**) average cross-sectional area (CSA) of regenerating myofibers and (**g**) number of myofibers containing two or more centrally located nuclei per field at day 5 post injury. *N*=6 in each group. (**h**) Protein levels of TAK1 and TAB1 in uninjured and injured TA muscle of TAK1^f/f^ and TAK1^scko^ mice at day 5 post injury. Error bars represent s.d. *P<0.05, values significantly different from corresponding injured TA muscle of TAK1^f/f^ mice by unpaired *t*-test. #*P*<0.05, values significantly different from 5d-injured TA muscle of TAK1^f/f^ mice by paired *t*-test. d, days.

We first investigated whether TAK1 is activated in satellite stem cells in adult animals. A combination of cell surface markers (CD45^−^, CD31^−^, Ter119^−^, Sca1^−^ and α7-integrin^+^) is widely used to identify satellite cells in adult mouse skeletal muscle^6^. We used a fluorescence-activated cell sorting (FACS)-based intracellular protein detection assay to examine the activation of TAK1 in satellite cells similar to as described^35^. Single-cell suspension prepared from hind limb muscle of wild-type (WT) mice were analysed by FACS for the expression of α7-integrin and phosphorylated form of TAK1 (Thr184/187). This analysis showed that phospho-TAK1 protein is expressed in the α7-integrin^+^ cells in satellite stem cells of naive WT mice (Fig. 1a; Supplementary Fig. 1A). We also investigated whether the phosphorylation TAK1 in satellite cells changes during muscle injury. Barium chloride (BaCl_2_) is a commonly used chemical agent that causes muscle depolarization and myofiber death by stimulating exocytosis while blocking the Ca^2+^ efflux^45^. BaCl_2_ produces uniform and reproducible muscle injury and does not affect satellite and stromal cells and hence the regeneration of the treated muscle(s) proceeds normally. Tibial anterior (TA) muscle of WT mice was injected with 100 μl of 1.2% BaCl_2_ and after 5 days, the level of phosphorylated TAK1 in satellite cells was detected by FACS. Our analysis showed that the amount of phosphorylated TAK1 (Thr184/187) was significantly increased in satellite cells upon skeletal muscle injury (Supplementary Fig. 1B,C).

To understand the role of TAK1, we inactivated TAK1 in satellite cells of adult mice. The second exon of the TAK1 gene (which encodes the enzyme's catalytic domain) is flanked with two loxp sites in the floxed TAK1 (henceforth, TAK1^f/f^) mice^36^ and thus can be deleted by Cre-mediated recombination. Pax7^CreER^ mice are tamoxifen-inducible satellite cell-specific Cre mouse line in which Cre expression can be induced at any time after birth by intraperitoneal injection of tamoxifen in mice^46^. We crossed TAK1^f/f^ mice with Pax7^CreER^ mice to generate satellite cell-specific inducible TAK1 knockout (Pax7^CreER^;TAK1^f/f^, henceforth, TAK1^scko^) mice (Supplementary Fig. 1D). Eight-week-old TAK1^scko^ mice were injected with tamoxifen to induce the TAK1 deletion. Littermate TAK1^f/f^ mice were also treated with tamoxifen and served as a control. The mice were maintained on a tamoxifen-containing chow for the entire duration of the experiment (Supplementary Fig. 1E). TA muscle of TAK1^scko^ and littermate TAK1^f/f^ mice were given intramuscular injection of 100 μl of 1.2% BaCl_2_ and regeneration was analysed at 5 and 14 days post injury. Inactivation of TAK1 in satellite cells did not affect the overall body weight (Fig. 1b) or wet weight of uninjured TA muscle (Fig. 1c). However, the wet weight of injured TA muscle was significantly reduced in TAK1^scko^ mice compared with TAK1^f/f^ mice (Fig. 1d). We next analysed injured TA muscle through performing haematoxylin and eosin (H&E) staining. Interestingly, regeneration of TA muscle was markedly reduced in TAK1^scko^ mice compared with the TA muscle of TAK1^f/f^ littermates at 5 days post injury (Fig. 1e). Although newly formed centrally nucleated fibres populated regenerating TA muscle of TAK1^f/f^ mice, the corresponding TAK1^scko^ muscle showed poor signs of regeneration evident by reduced number and size of centrally nucleated fibres with robust persistence of cellular infiltrate. Morphometric analyses of 5-day-injured TA muscle sections showed ∼40% reduction in the average fibre cross-sectional area (CSA) in TAK1^scko^ mice compared with TAK1^f/f^ littermates (Fig. 1f). Moreover, the number of fibres containing two or more centrally located nuclei was also significantly reduced in TAK1^scko^ mice further indicating deficit in muscle regeneration (Fig. 1g). Reduced regeneration of TA muscle of TAK1^scko^ mice was also evident at 14 days after muscle injury (Fig. 1e). Western blot analysis revealed that TAK1 protein levels were reduced by ∼50% in 5-day-injured TA muscle of TAK1^scko^ mice compared with TAK1^f/f^ mice (Fig. 1h). Moreover, the levels of TAK1-binding protein, TAB1, were also found to be considerably reduced in injured muscle of TAK1^scko^ mice (Fig. 1h). These results suggest that TAK1 in satellite cells is essential for skeletal muscle regeneration.

### Inactivation of TAK1 inhibits regenerative myogenesis in mice

**Figure 2:**
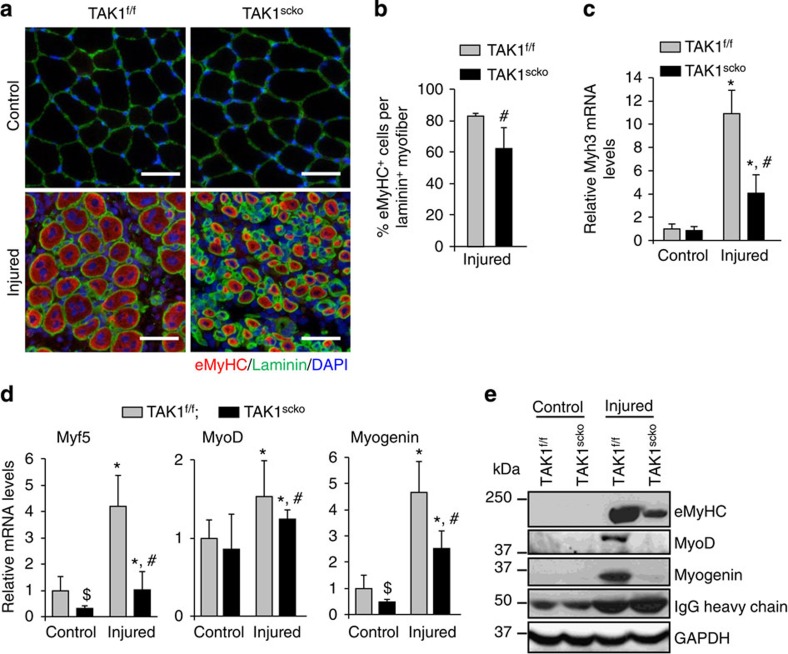
Inactivation of TAK1 in satellite cells inhibits formation of new myofibers and expression of myogenic regulatory factors following injury. (**a**) Representative photomicrographs of uninjured and 5d-injured TA muscle section of TAK1^f/f^ and TAK1^scko^ mice after immunostaining for eMyHC (red colour) and laminin (green). Nuclei were identified by staining with DAPI. Scale bar, 100 μm. (**b**) Percentage of eMyHC^+^ fibres per laminin^+^ myofiber in 5d-injured TA muscle of TAK1^f/f^ and TAK1^scko^ mice. *N*=6 in each group for eMyHC and laminin staining. Relative mRNA levels of (**c**) eMyHC (that is, Myh3) and (**d**) Myf5, MyoD and myogenin in uninjured and 5d-injured TA muscle of TAK1^f/f^ and TAK1^scko^ mice measured by qRT-PCR assay. *N*=3 in each group for qRT-PCR analysis. (**e**) Representative immunoblots presented here demonstrate the protein levels of MyoD, myogenin, eMyHC, IgG and GAPDH in uninjured and injured muscle of TAK1^f/f^ and TAK1^scko^ mice. Error bars represent s.d. *P<0.05, values significantly different from corresponding control TA muscle of TAK1^f/f^ or TAK1^scko^ mice by unpaired *t*-test. #*P*<0.05, values significantly different from corresponding injured TA muscle of TAK1^f/f^ mice by paired *t*-test. $*P*<0.05, values significantly different from control TA muscle of TAK1^f/f^ mice by paired *t*-test. d, days.

Regeneration of myofibers upon injury involves hierarchical expression of myogenic regulatory factors Myf5, MyoD, and myogenin and embryonic/developmental myosin heavy chain (eMyHC)^5^,^47^. There was no detectable expression of eMyHC in uninjured TA muscle of either genotype (Fig. 2a). By contrast, the expression of eMyHC was clearly evident on the 5-day-injured TA muscle sections. Interestingly, a more uniform and abundant expression of eMyHC was evident in TA muscle of TAK1^f/f^ mice compared with TAK1^scko^ mice (Fig. 2a). The size and number of eMyHC^+^ fibres were significantly reduced in regenerating TA muscle of TAK1^scko^ mice compared with corresponding TAK1^f/f^ mice (Fig. 2a,b). Moreover, messenger RNA (mRNA) levels of eMyHC (gene name: *Myh3*) were significantly reduced in injured muscle of TAK1^scko^ mice compared with TAK1^f/f^ mice (Fig. 2c). Furthermore, we found that the mRNA levels of Myf5, MyoD and myogenin were significantly reduced in injured TA muscle of TAK1^scko^ mice compared with injured TA muscle of TAK1^f/f^ mice (Fig. 2d). Surprisingly, a significant decrease in the mRNA levels of Myf5 and myogenin was noticeable in uninjured muscle of TAK1^scko^ mice compared with TAK1^f/f^ mice (Fig. 2d). Western blot analysis also showed that the protein levels of eMyHC, MyoD and myogenin were drastically reduced in injured muscle of TAK1^scko^ mice compared with TAK1^f/f^ mice (Fig. 2e). These results suggest that TAK1 in satellite stem cells plays a pivotal role in mediating the early stages of myogenesis in regenerating myofibers.

### Disruption of TAK1 reduces satellite cells pool in skeletal muscle

**Figure 3:**
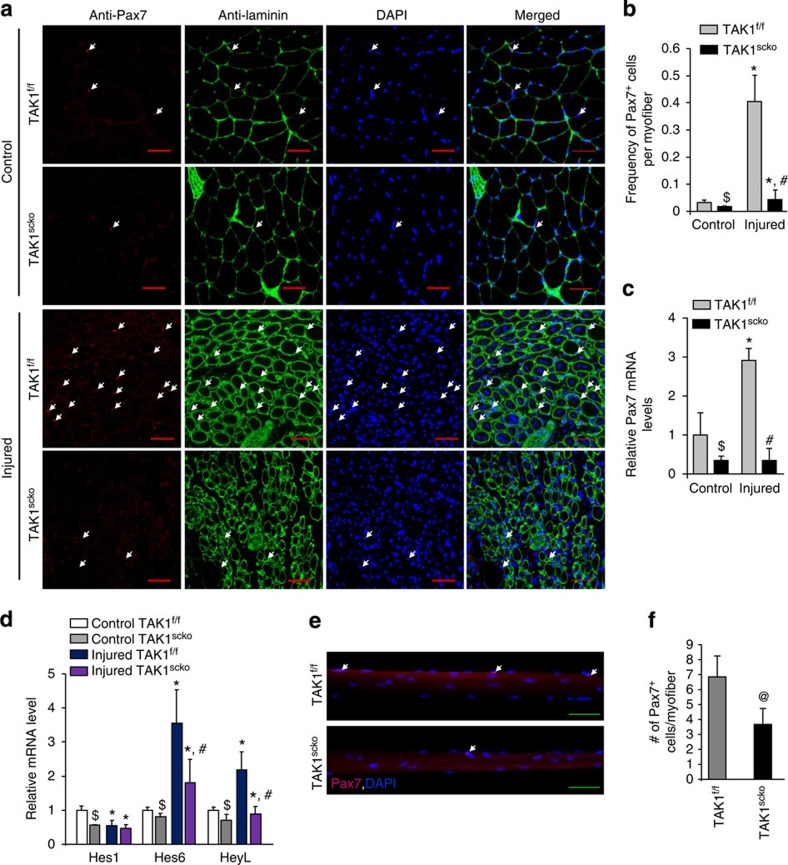
TAK1 is required for the maintenance of satellite cell pool in skeletal muscle. TA muscle of TAK1^f/f^ and TAK1^scko^ mice were injected with 1.2% BaCl_2_ or saline alone for 5d. The muscles were isolated and processed for histological and biochemical analysis. (**a**) Representative photomicrographs of Pax7-stained sections. Nuclei were identified by co-staining with DAPI. Arrows point to Pax7^+^ cells. Scale bar, 100 μm. (**b**) Quantification of frequency of Pax7^+^ cells per myofiber in uninjured and injured muscle of TAK1^f/f^ and TAK1^scko^ mice. *N*=4 in each group for quantification of Pax7^+^ cells in uninjured and injured myofibers. (**c**) Relative mRNA of Pax7 in uninjured and 5d-injured TA muscle of TAK1^f/f^ and TAK1^scko^ mice measured by performing qRT-PCR. (**d**) Relative mRNA levels of Notch target genes Hes1, Hes6 and HeyL in uninjured and injured TA muscle of TAK1^f/f^ and TAK1^scko^ mice. *N*=4 in each group for qRT-PCR assays. (**e**) Single myofibers were isolated from EDL muscle of TAK1^f/f^ and TAK1^scko^ mice and immediately fixed and labelled with antibodies against Pax7. Nuclei were counterstained with DAPI. Representative merged images of freshly isolated myofibers from TAK1^f/f^ and TAK1^scko^ mice stained with Pax7 and DAPI. Scale bar, 100 μm. (**f**) Quantification of number of Pax7^+^ cells per myofibers. *N*=3 mice in each group. A total of 18–20 myofibers were included for quantification from each mouse. Error bars represent s.d. *P<0.05, values significantly different from corresponding control TA muscle of TAK1^f/f^ or TAK1^scko^ mice by paired *t*-test. #*P*<0.05, values significantly different from corresponding injured TA muscle of TAK1^f/f^ mice by paired *t*-test. $*P*<0.05, values significantly different from control TA muscle of TAK1^f/f^ mice by paired *t*-test. @*P*<0.05, values significantly different from TAK1^f/f^ cultures by unpaired *t*-test. d, days.

Transcription factor Pax7, which is expressed in both quiescent and activated satellite cells, is critical for maintaining satellite cell function^48^,^49^. Results showed that the frequency of Pax7^+^ cells was significantly reduced in TA muscle of TAK1^scko^ mice compared with TAK1^f/f^ mice 5 days after injury (Fig. 3a,b). Remarkably, we also found that the frequency of Pax7^+^ cells within basal lamina was significantly reduced even in naive TA muscle of TAK1^scko^ mice compared with littermate TAK1^f/f^ mice (Fig. 3a,b). Consistent with immunohistochemistry results, mRNA levels of Pax7 were also found to be significantly downregulated in uninjured muscle of TAK1^scko^ mice. About threefold upregulation in the mRNA levels of Pax7 was observed in TA muscle TAK1^f/f^ mice 5 days post injury, however, such upregulation was clearly absent in TAK1^scko^ mice (Fig. 3c).

Notch signalling is an important regulator of satellite cell dynamics in both quiescent and activated states. Notch target genes such as Hes1 and HeyL are involved in maintaining the quiescent status of satellite cells^7^. A significant reduction in mRNA levels of Hes1, Hes6 and HeyL was observed in 5-day-injured TA muscle of TAK1^scko^ mice compared with corresponding muscle of TAK1^f/f^ mice (Fig. 3d). Intriguingly, the mRNA levels of Hes1, Hes6 and HeyL were also found to be significantly reduced in uninjured TA muscle of TAK1^scko^ mice compared with TAK1^f/f^ mice (Fig. 3d). We also investigated whether the deletion of TAK1 in satellite cells affect the expression of certain inflammatory cytokines in injured muscle microenvironment. However, there was no significant difference in the transcript levels of tumour necrosis factor-α (TNF)-α or its receptors, TWEAK, and IL-6 between injured TA muscle of TAK1^f/f^ and TAK1^scko^ mice (Supplementary Fig. 2A).

We also prepared single-myofiber cultures from extensor digitorum longus (EDL) muscle of TAK1^f/f^ and TAK1^scko^ mice. Immediately after isolation, the cultures were fixed with paraformaldehyde (PFA) and myofiber-associated cells were immunostained for Pax7 and enumerated (Fig. 3e). Consistent with above results, the number of Pax7^+^ cells per myofiber was significantly reduced in TAK1^scko^ cultures compared with TAK1^f/f^ mice (Fig. 3f). Taken together, these results suggest that TAK1 is essential for preserving the pool of satellite stem cells in adult skeletal muscle.

### TAK1 is required for the proliferation of satellite stem cells

**Figure 4:**
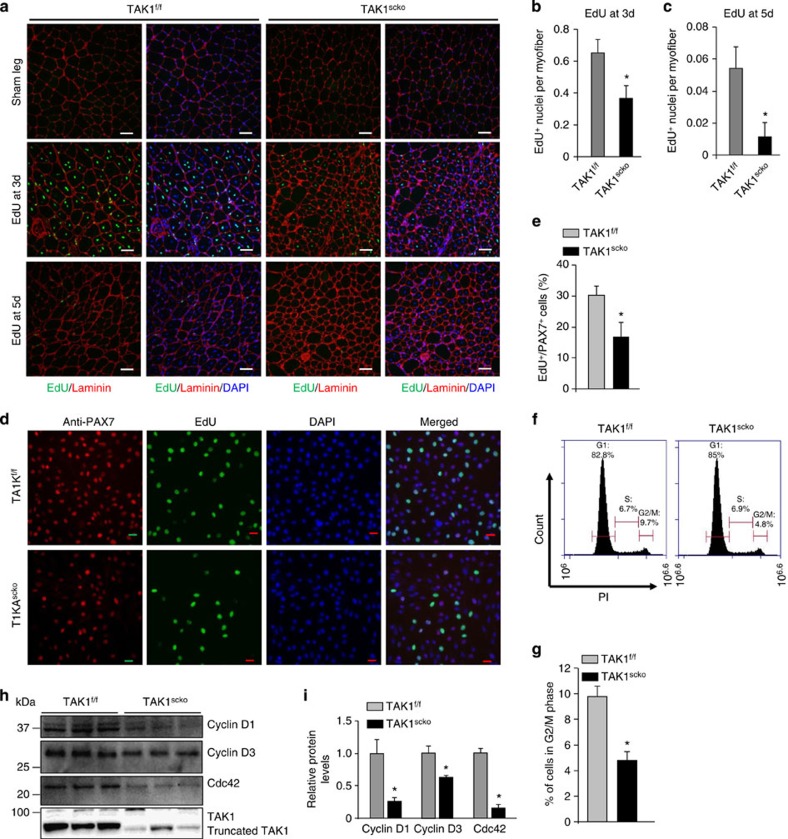
TAK1 is required for the proliferation of satellite cells. TA muscle of TAK1^f/f^ and TAK1^scko^ mice were given intramuscular injection of saline alone or 1.2% BaCl_2_ solution. At the day 3 or day 5, the mice were given intraperitoneal injection of EdU. The TA muscle was isolated at day 14 post injury and analysed by staining for EdU, anti-laminin and DAPI. (**a**) Representative merged images of control and injured TA muscle sections of TAK1^f/f^ and TAK1^scko^ mice. Quantitative estimation of number of EdU^+^ nuclei per myofiber in TAK1^f/f^ and TAK1^scko^ mice injected with EdU at (**b**) day 3 or (**c**) day 5 post injury. *N*=3 in each group for EdU incorporation assay and quantification. (**d**) Primary myogenic cultures were established from non-tamoxifen-treated TAK1^f/f^ and TAK1^scko^ mice. The cells were incubated with 4-hydroxytamoxifen (TAM) for 48 h to induce Cre-mediated recombination. The cells were then washed and cultured for additional 48 h. EdU was added in the culture to label proliferating cells during last 90 min. The cells were then fixed and stained with anti-Pax7, EdU and DAPI. Representative individual and merged images of TAK1^f/f^ and TAK1^scko^ cultures after staining for Pax7, EdU and DAPI. (**e**) Quantitative estimation of percentage of Pax7^+^/EdU^+^ double-positive cells in TAK1^f/f^ and TAK1^scko^ cultures. (**f**) Cell cycle analysis of TAK1^f/f^ and TAK1^scko^ cultures by FACS method. Representative histograms presented here show distribution of cells in G1, S and G2/M phases of cell cycle. (**g**) Quantification of proportion of cells in G2/M phase of cell cycle in TAK1^f/f^ and TAK1^scko^ cultures. *N*=5–6 in each group for Pax7 staining and EdU incorporation and cell cycle analysis. (**h**) Immunoblots presented here demonstrate relative levels of cyclin D1, cyclin D3 and Cdc42, and full-length and truncated TAK1 in TAK1^f/f^ and TAK1^scko^ cultures. (**i**) Densitometry analysis of immunoblots for cyclin D1, cyclin D3 and Cdc42 levels in TAK1^f/f^ and TAK1^scko^ cells. *N*=3 for western blot and densitometry analysis. Error bars represent s.d. *P<0.05 values significantly different from corresponding TAK1^f/f^ mice or TAK1^f/f^ myogenic cultures by unpaired *t*-test. d, days.

To understand why inactivation of TAK inhibits muscle regeneration, we next investigated the role of TAK1 in the proliferation of satellite cells in response to muscle injury. TA muscle of TAK1^f/f^ and TAK1^scko^ mice were injured by intramuscular injection of 1.2% BaCl_2_ solution. The mice were given intraperitoneal injection of 5-ethynyl-2′-deoxyuridine (EdU) at day 3 or day 5 and the TA muscle was isolated at day 14 post-BaCl_2_ injection. The muscle sections made were stained for EdU and anti-laminin, whereas 4,6-diamidino-2-phenylindole (DAPI) staining was used to label nuclei. The number of EdU^+^ nuclei within the myofibers was studied. The number of EdU^+^ nuclei was drastically increased in injured muscle especially when the EdU was injected on day 3 or 5 post injury (Fig. 4a). Interestingly, the number of EdU^+^ nuclei per myofiber was found to be significantly reduced in TAK1^scko^ mice compared with TAK1^f/f^ mice (Fig. 4b,c). It is notable that injured TA muscle of TAK1^scko^ mice contains many centrally placed nuclei, which have not been marked by EdU, at either day 3 or day 5 of administration of EdU in mice post injury (Fig. 4a). These nuclei may have arisen by proliferation of the minority of remaining satellite cells in TAK1^scko^ mice. It is also possible that EdU, like other thymidine analogue markers, is diluted by progressive mitosis to the point of undetectability. Therefore, the lower frequency of EdU^+^ nuclei in the injured TA muscle of TAK1^scko^ mice is a result of diminished proliferation or excessive proliferation that has diluted the EdU to below detectability.

Consistent with *in vivo* results, we found that the number of EdU^+^-positive satellite cells per myofiber was significantly reduced in myofiber cultures established from EDL muscle of TAK1^scko^ mice compared with TAK1^f/f^ both at 24 and 72 h of culture (Supplementary Fig. 3A,B).

We next investigated the role of TAK1 in proliferation of myofiber-free cultured satellite cells. Satellite cells from hind limb muscle of non-tamoxifen-treated TAK1^f/f^ and TAK1^scko^ mice were isolated and expanded in cultures followed by treatment with 4-hydroxytamoxifen (TAM) for 48 h to induce tamoxifen-mediated Cre recombination. PCR analysis using genomic DNA from these cells confirmed that treatment with TAM efficiently truncated the kinase domain in TAK1^scko^ cells (Supplementary Fig. 1F). After 48 h of removal of TAM, the proliferation of Pax7^+^ cells was measured by pulse labelling with EdU. Results showed that the inactivation of TAK1 significantly reduced the proliferation of Pax7^+^ cells (Fig. 4d,e). Using a FACS-based approach, we also performed cell cycle analysis and found that the proportion of cells in G2/M phase of cell cycle was significantly reduced in TAK1^scko^ cultures compared with TAK1^f/f^ cultures indicating cell cycle arrest in TAK1^scko^ cultures (Fig. 4f,g). Furthermore, protein levels of cyclin D1, cyclin D3 and Cdc42 protein, which are known to promote cellular proliferation, were significantly reduced in TAK1^scko^ cultures compared with TAK1^f/f^ cultures (Fig. 4h,i). Moreover, we found that the kinase domain of TAK1 was efficiently deleted (>95% loss in WT TAK1 protein) in TAK1^scko^ cultures upon treatment with TAM (Fig. 4h).

We also studied the effect of pharmacological inhibition of TAK1 on satellite cell proliferation *in vitro*. Primary myogenic cultures prepared from WT mice were treated with vehicle alone or 5Z-7-oxozeaenol (5Z7O), a pharmacological inhibitor of TAK1 (ref. 50), for 24 h and the cellular proliferation of Pax7^+^ cells was assayed by studying the incorporation of EdU. Treatment with 5Z7O drastically reduced the incorporation of EdU in Pax7^+^ cells (Supplementary Fig. 4A,B). Collectively, these results suggest that TAK1 is required for the proliferation of satellite cells both *in vivo* and *in vitro*.

### TAK1 prevents precocious differentiation of satellite cells

**Figure 5:**
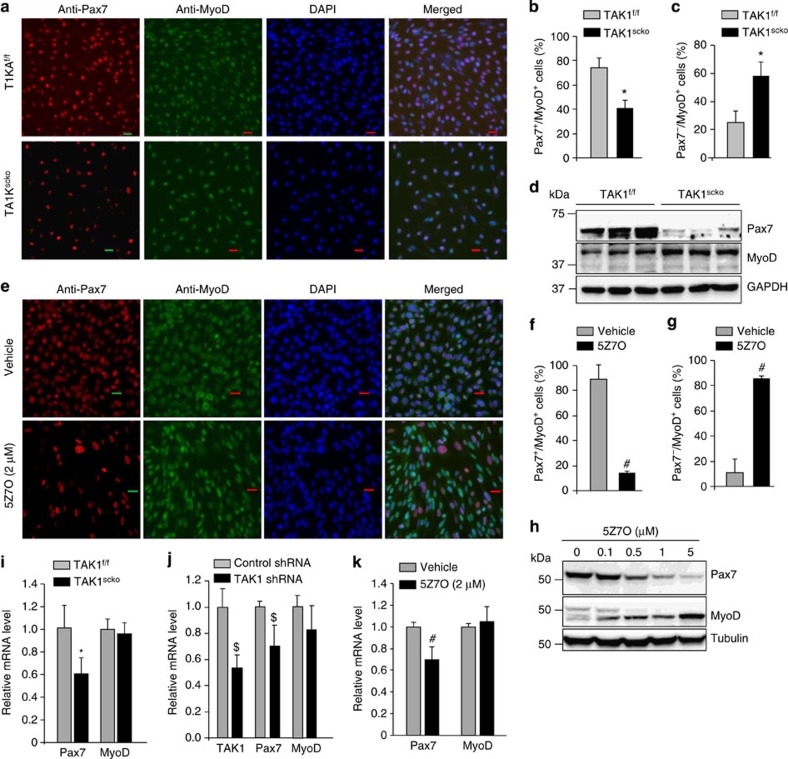
Inactivation of TAK1 reduces the proportion of Pax7^+^ cells in myogenic cultures. (**a**) Primary myogenic cultures were established from TAK1^f/f^ and TAK1^scko^ mice without treatment with tamoxifen. The cells were treated with 4-hydoxytamoxifen (TAM) for 48 h to induce Cre-mediated recombination. The cells were then washed and cultured for additional 48 h followed by staining with anti-Pax7, anti-MyoD and DAPI. Representative individual staining for Pax7, MyoD, and DAPI and merged images are presented here. Scale bar, 20 μm. Quantitative estimation of percentage of (**b**) Pax7^+^/MyoD^+^ and (**c**) Pax7^−^/MyoD^+^ cells (normalized with DAPI) in TAK1^f/f^ and TAK1^scko^ cultures. (**d**) Western blot analysis of Pax7 and MyoD levels in TAK1^f/f^ and TAK1^scko^ cultures. (**e**) Primary myogenic cultures established from WT mice were treated with vehicle alone or 2 μM 5Z-7-oxozeaenol (5Z7O) for 24 h followed by staining for Pax7, MyoD and nuclei (that is, DAPI). Representative individual staining for Pax7, MyoD, and DAPI and merged images are presented here. Scale bar, 20 μm. Percentage of (**f**) Pax7^+^/MyoD^+^ cells and (**g**) Pax7^−^/MyoD^+^ cells (normalized with DAPI) in control and 5Z7O-treated cultures. (**h**) Primary myogenic cultures of WT mice were treated with indicated concentration of 5Z7O for 24 h followed by immunoblotting for Pax7 and MyoD. Representative immunoblots are presented here. (**i**) Relative mRNA levels of Pax7 and MyoD in TAK1^f/f^ and TAK1^scko^ cultures measured by performing qRT-PCR assay. (**j**) Primary myogenic cells were transfected with control shRNA or TAK1 shRNA for 36 h followed by measuring mRNA levels of TAK1, Pax7 and MyoD. (**k**) Primary myogenic cells prepared from WT mice were treated with vehicle alone or 2 μM 5Z7O for 6 h and the mRNA levels of Pax7 and MyoD were measured by qRT-PCR assay. *N*=4 in each group for immunostaining and quantification experiment, whereas *N*=3 for qRT-PCR and western blot analysis. Error bars represent s.d. *P<0.05 values significantly different from TAK1^f/f^ cultures by unpaired *t*-test. #*P*<0.05, values significantly different from the cultures treated with vehicle alone by unpaired *t*-test. $*P*<0.05, values significantly different from corresponding cultures transfected with control shRNA by unpaired *t*-test.

The expression pattern of Pax7 and MyoD specifies the myogenic status of satellite cells as quiescent (Pax7^+^/MyoD^−^), activated (Pax7^+^/MyoD^+^) or differentiated (Pax7^−^/MyoD^+^)^3^,^6^. We next investigated whether TAK1 has any role in maintenance of myogenic potential of satellite cells. Satellite cells isolated from hind limb muscle of non-tamoxifen-treated TAK1^f/f^ and TAK1^scko^ mice were treated with TAM for 48 h. The cells were then washed and incubated in fresh growth medium without TAM for additional 48 h followed by immunostaining for Pax7 and MyoD proteins. All Pax7^+^ cells also expressed MyoD, which is typical of cultured proliferating satellite cells. Interestingly, the number of Pax7^+^/MyoD^+^ cells was found to be significantly reduced, whereas the number of Pax7^−^/MyoD^+^ was significantly increased in TAK1^scko^ cultures compared with corresponding TAK1^f/f^ cultures (Fig. 5a–c). Consistent with immunostaining results, the levels of Pax7 protein were significantly reduced, whereas the levels of MyoD protein were significantly increased in TAK1^scko^ cultures (Fig. 5d; Supplementary Fig. 5A). Moreover, we found that pharmacological inhibition of TAK1 using 5Z7O also drastically reduced the number of Pax7^+^/MyoD^+^ and enhanced the number of Pax7^−^/MyoD^+^ cells in myogenic cultures from WT mice (Fig. 5e–g). Treatment with 5Z7O also reduced the levels of Pax7 protein and increased the levels of MyoD protein in myogenic cultures from WT mice in a dose-dependent manner (Fig. 5h).

Although we found changes in Pax7 and MyoD protein levels, it remained unknown whether TAK1 regulates their expression in satellite cells. To address this question, we measured transcript levels of Pax7 and MyoD in TAK1^f/f^ and TAK1^scko^ cultures. Interestingly, the mRNA levels of Pax7 were significantly reduced in TAK1^scko^ cultures compared with TAK1^f/f^ cultures. However, there was no significant difference in the mRNA levels of MyoD between TAK1^f/f^ and TAK1^scko^ satellite cells (Fig. 5i). Moreover, transfection of WT satellite cell cultures with TAK1 short hairpin RNA (shRNA) significantly reduced the mRNA levels of Pax7 without having any effect on MyoD mRNA (Fig. 5j). The mRNA levels of Pax7 (but not MyoD) were also significantly reduced within 6 h of treatment of WT satellite cells with 5Z7O (Fig. 5k). Taken together, these results suggest that TAK1 is required for maintaining the satellite cell self-renewal/proliferation capacity and preventing precocious progression in myogenic lineage.

### TAK1 is required for the survival of satellite cells

**Figure 6:**
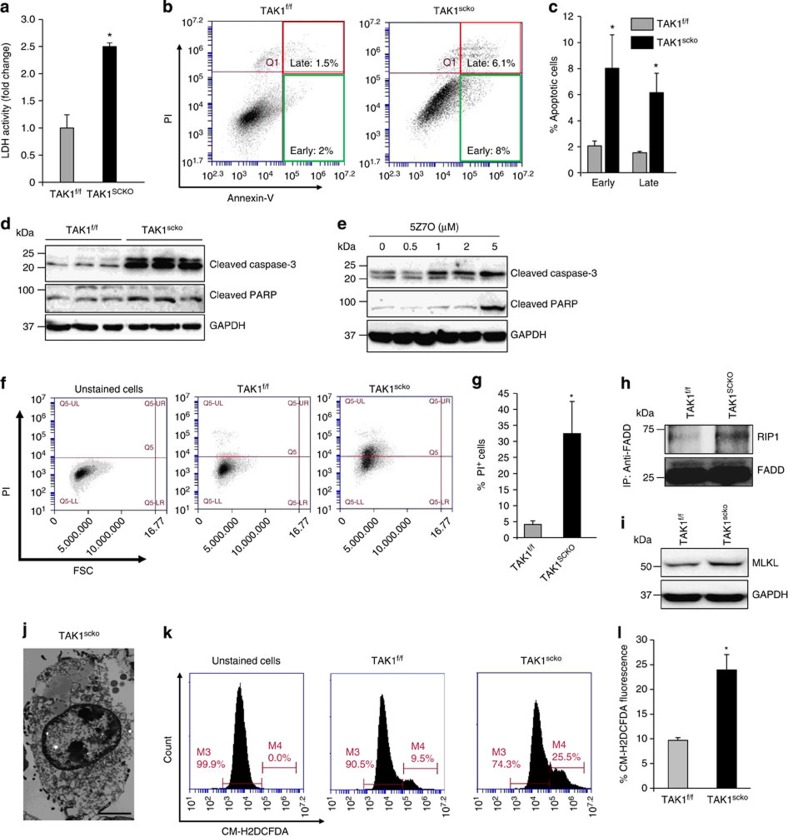
Inactivation of TAK1 induces cell death in satellite cell cultures. Primary myogenic cultures were established from non-tamoxifen-treated TAK1^f/f^ and TAK1^scko^ mice and the cells were then treated with 4-hydoxytamoxifen (TAM) for 48 h to induce Cre-mediated recombination. After washing the cells, they were incubated in growth medium for another 72 h. (**a**) Relative amounts of lactate dehydrogenase (LDH) in supernatants of TAK1^f/f^ and TAK1^scko^ cultures. (**b**) The cells were collected and stained for Annexin V and propidium iodide (PI) and analysed by FACS to detect early and late apoptotic cells. Representative dot plots are presented here. (**c**) Quantification of early and late apoptotic cells in TAK1^f/f^ and TAK1^scko^ cultures after FACS analysis. (**d**) Immunoblots presented here demonstrate relative levels of cleaved caspase-3 and cleaved poly (ADP-ribose) polymerase (PARP) in TAK1^f/f^ and TAK1^scko^ cultures. (**e**) Primary myogenic cultures prepared from WT mice were treated with indicated concentrations of 5Z-7-oxozeaenol (5Z7O) for 24 h followed by performing western blotting for cleaved capsase-3, cleaved PARP and an unrelated protein GAPDH. Representative immunoblots from two independent experiments are presented here. (**f**) After the removal of TAM and culturing for 72 h, the TAK1^f/f^ and TAK1^scko^ cells were stained for PI and analysed by FACS to detect necroptosis. Representative dot plots of FACS analysis of unstained and PI^+^ cells in TAK1^f/f^ and TAK1^scko^ cultures are presented here. (**g**) Quantification of percentage of PI^+^ cells in TAK1^f/f^ and TAK1^scko^ cultures. (**h**) TAK1^f/f^ and TAK1^scko^ cells were immunoprecipitated with anti-FADD followed by immunoblotting to detect RIP1 and FADD protein. Immunoblots presented here demonstrate increased interaction of FADD with RIP1 in TAK1^scko^ cultures. (**i**) Immunoblot presented here demonstrates levels of MLKL protein in TAK1^f/f^ and TAK1^scko^ cultures. (**j**) Representative image of transmission electron microscopy showing necroptosis in TAK1^scko^ cell. Scale bar, 2 μm. (**k**) TAK1^f/f^ and TAK1^scko^ cells were incubated with CM-H2DCFDA dye for 20 min followed by analysis with FACS. (**l**) Quantification of cells stained positive with CM-H2DCFDA. *N*=3–4 in each group for all the experiments in this figure. Error bars represent s.d. *P<0.05 values significantly different from TAK1^f/f^ cultures by unpaired *t*-test.

Published reports suggest that TAK1 is a critical regulator for survival of many cell types^21^,^34^,^41^. Since the number of satellite cells (Pax7^+^) was reduced in TAK1^scko^ mice, we next sought to investigate whether disruption of TAK1 also affects the survival of satellite cells. Lactate dehydrogenase (LDH) is a stable cytoplasmic enzyme present in all cells and rapidly released into the cell culture supernatant upon damage of the plasma membrane. Satellite cells were isolated from hind limb muscle of non-tamoxifen-treated TAK1^f/f^ and TAK1^scko^ mice and expanded in cultures followed by treatment with TAM for 48 h after which the cells were washed and incubated in fresh growth medium without TAM. There was no significant difference in the cellular viability immediately after the removal of TAM. However, a drastic increase in cell death was noticeable after 72 h of TAM removal in TAK1^scko^ cultures, which was confirmed by measuring the levels of LDH in culture supernatants (Fig. 6a). To understand the mode of cell death, we first studied apoptosis by performing AnnexinV/propidium iodide (PI) staining followed by analysis with FACS. Results showed that there was a significant increase in both early (TAK1^f/f^ versus TAK1^scko^: ∼2 versus 8%) and late (TAK1^f/f^ versus TAK1^scko^, ∼1.5 versus 6%) apoptotic cells in TAK1^scko^ cultures compared with TAK1^f/f^ cultures (Fig. 6b,c). Western blot analysis of cellular extracts showed a significant increase in the levels of cleaved caspase-3 and poly (ADP-ribose) polymerase protein, the markers of apoptosis, in TAK1^scko^ cultures (Fig. 6d; Supplementary Fig. 5B). Moreover, pharmacological inhibition of TAK1 activity using 5Z7O also increased the levels of cleaved capsase-3 and cleaved poly (ADP-ribose) polymerase in satellite cell cultures (Fig. 6e).

Although there was significant increase in the proportion of apoptotic cells in TAK1^scko^ cultures, it does not account for the substantial cell death that occurs in TAK1^scko^ cultures after 72–96 h of the removal of TAM. Accumulating evidence suggests that disruption of TAK1 can also induce necroptosis, a recently identified programed cell death, in some other cell types^34^,^40^,^41^. Necroptosis can be detected by performing PI staining on the cells^41^. Therefore, we next stained the TAK1^f/f^ and TAK1^scko^ cultures with PI and performed FACS analysis. Interestingly, there was a marked increase in the number of PI^+^ cells in TAK1^scko^ cultures compared with TAK1^f/f^ cultures (Fig. 6f,g). Cell death through necroptosis involves the formation of RIP1, Fas-associated death domain (FADD) and RIP3 necrosome complex^38^,^39^. To detect the RIP1–RIP3–FADD necrosome, the cell extracts made were immunoprecipitated with anti-FADD, followed by immunoblotting with anti-RIP1. As shown in Fig. 6h, there was a considerable increase in the association of RIP1 with FADD in TAK1^scko^ cultures compared with TAK1^f/f^ cultures. It has also been reported that in response to specific stimuli, RIP3 phosphorylates mixed lineage kinase domain-like protein (MLKL) leading to the progression of necroptosis. Levels of both total and phosphorylated MLKL protein are increased in the cells undergoing cell death through necroptosis^39^,^51^. Our analysis showed that the protein levels of MLKL are considerably increased in TAK1^scko^ cells compared with corresponding TAK1^f/f^ cells (Fig. 6i). Finally, we performed transmission electron microscopy analysis that revealed typical features of necroptosis such as early plasma membrane permeabilization, translucent cytosol and swollen cell organelles in TAK1^scko^ cells (Fig. 6j).

Increased levels of reactive oxygen species (ROS) can trigger cell death through apoptosis or necroptosis^42^,^52^,^53^. Using CM-H2DCFDA fluorescent dye and FACS technique, we next compared the levels of ROS in TAK1^f/f^ and TAK1^scko^ satellite cells cultures. Interestingly, a drastic increase in ROS was observed in TAK1^scko^ cultures compared with TAK1^f/f^ cultures (Fig. 6k,l).

### Nec-1 improves survival and proliferation of TAK1^−/−^ cells

**Figure 7:**
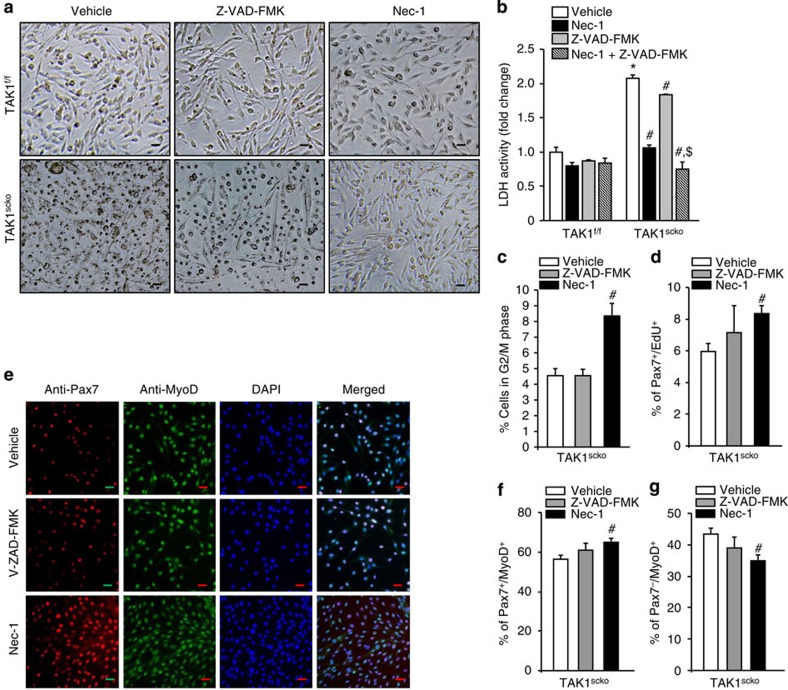
Nec-1 improves survival and proliferation of TAK1^scko^ cells. Primary myogenic cells isolated from non-tamoxifen-treated TAK1^f/f^ and TAK1^scko^ mice were incubated with TAM in medium containing vehicle alone (DMSO), 20 μM z-VAD-FMK, or 20 μM Nec-1 for 48 h. The cells were then washed and incubated in growth medium containing vehicle alone (DMSO), 20 μM z-VAD-FMK, or 20 μM Nec-1 and analysed at specific time points. (**a**) Representative phase contrast images of TAK1^f/f^ and TAK1^scko^ cultures taken after 72 h of removal of TAM. Scale bar, 20 μm. (**b**) Amounts of LDH in culture supernatants TAK1^f/f^ and TAK1^scko^ cells treated with vehicle alone, z-VAD-FMK (20 μM), Nec-1 (20 μM) or both z-VAD-FMK (20 μM) and Nec-1 (20 μM) measured after 72 h of removal of TAM. (**c**) Percentage of cells in G2/M phase of cell cycles at 72 h after removal of TAM in TAK1^scko^ cultures assayed by FACS analysis. (**d**) Percentage of Pax7^+^/EdU^+^ cells in TAK1^scko^ cultures treated with z-VAD-FMK or Nec-1 assayed after 48 h of removal of TAM. (**e**) Representative individual staining for Pax7, MyoD, and DAPI and merged images of TAK1^scko^ cultures incubated with vehicle alone, z-VAD-FMK or Nec-1 peptide and after 48 h of removal of TAM. Scale bar, 20 μm. Quantification of percentage of (**f**) Pax7^+^/MyoD^+^ and (**g**) Pax7^−^/MyoD^+^ cells in TAK1^scko^ cultures incubated with vehicle alone, z-VAD-FMK or Nec-1 peptide. *N*=4 in each group in all the experiments in this figure. Error bars represent s.d. *P<0.01, values significantly different from corresponding TAK1^f/f^ cultures by unpaired *t*-test. #*P*<0.05, values significantly different from TAK1^scko^ cultures treated with vehicle alone by paired *t*- test. $*P*<0.01, values significantly different from TAK1^scko^ cultures treated with vehicle, z-VAD-FMK or Nec-1 alone by paired *t*-test. DMSO, dimethylsulphoxide.

To further understand whether the cell death in TAK1^scko^ cultures occurs by apoptosis or necroptosis, we studied the effects of Z-VAD-FMK (a pan-caspase peptide inhibitor) or necrostatin-1 (Nec-1, a peptide inhibitor of RIP1 kinase-dependent necroptosis) on cellular viability. Satellite cell cultures established from non-tamoxifen-treated TAK1^f/f^ and TAK1^scko^ mice were treated with TAM to induce Cre-mediated recombination in the presence of vehicle alone, Z-VAD-FMK, or Nec-1 for 48 h, followed by washing the cells and incubation in fresh growth medium along with Z-VAD-FMK or Nec-1 for additional 72 h. Treatment of z-VAD-FMK had only a small (but significant) effect towards improving the survival of TAK1^scko^ cells (Fig. 7a,b). By contrast, treatment with Nec-1 markedly improved cellular viability in TAK1^scko^ cultures suggesting that necroptosis is the major mechanism of cell death in satellite cells upon inactivation of TAK1 (Fig. 7a,b). Treatment with both Nec-1 and Z-VAD-FMK almost completely inhibited cell mortality in TAK1^scko^ cultures (Fig. 7b). Activation of RIP1 is also known to increase the production of TNF-α in some cell types^54^. By performing quantitative real-time PCR (qRT-PCR) assay, we investigated whether the expression of TNF-α is increased in TAK1^scko^ cells, which may cause cell death in an autocrine manner. Surprisingly, we found that the mRNA levels of TNF-α are significantly reduced in TAK1^scko^ cells compared with TAK1^f/f^ cells (Supplementary Fig. 2B). Moreover, incubation with TNF-α neutralizing antibody did not affect cell death in TAK1^scko^ cultures (Supplementary Fig. 2C) indicating that TNF-α is not the reason for the increased cell death in TAK1^scko^ cultures. We next studied the effect of z-VAD-FMK and Nec-1 on proliferation and the expression of Pax7 and MyoD in satellite cells. There was no significant effect of z-VAD-FMK or Nec-1 on any of these parameters in TAK1^f/f^ cells. However, we found that treatment with Nec-1 significantly increased the proportion of cells in G2/M phase of cell cycle (Fig. 7c) and increased the proliferation of Pax7^+^ cells assayed by EdU incorporation (Fig. 7d). Although a small increase in number of Pax7^+^/EdU^+^ cells was evident upon treatment of TAK1^scko^ cultures with z-VAD-FMK, it was not statistically significant (Fig. 7d).

We also performed immunostaining for Pax7 and MyoD on cells treated with z-VAD-FMK and Nec-1 peptide. Our analysis showed that proportion of Pax7^+^/MyoD^+^ cells was significantly increased, whereas the proportion of Pax7^−^/MyoD^+^ cells was significantly decreased in TAK1^scko^ cultures treated with Nec-1 compared with those treated with vehicle alone. By contrast, z-VAD-FMK had no significant effect on the number of Pax7^+^/MyoD^+^ or Pax7^−^/MyoD^+^ cells (Fig. 7e–g). These results suggest that while apoptosis may contribute to some extent, necroptosis is the major mechanism of satellite cell death upon inactivation of TAK1.

### TAK1 is required for the activation of NF-κB in satellite cells

**Figure 8:**
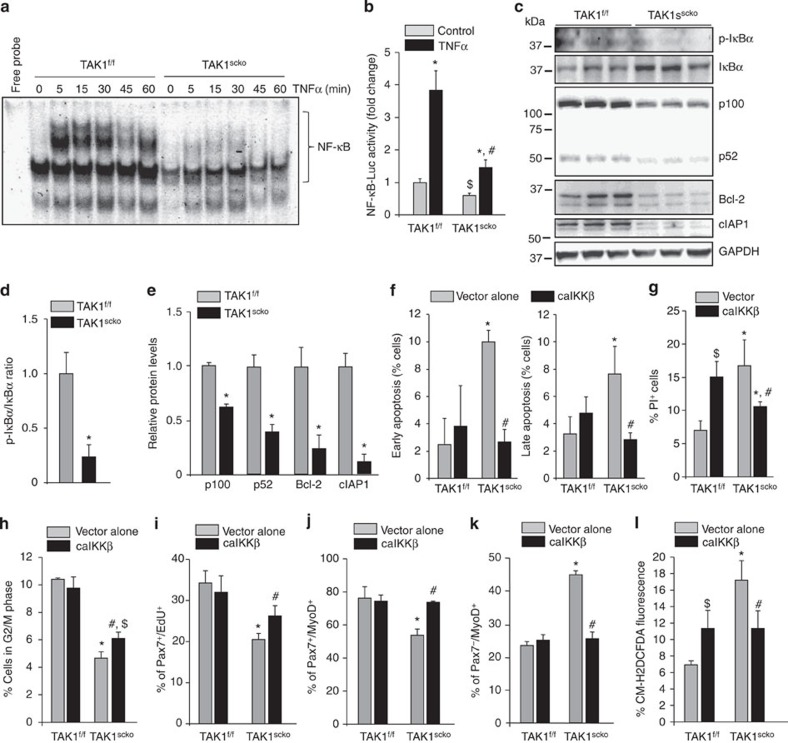
TAK1 is required for the activation of NF-κB in satellite cells. (**a**) TAK1^f/f^ and TAK1^scko^ cells were treated with 10 ng ml^−1^ TNFα for indicated time period and the nuclear extracts made were analysed by performing electrophoretic mobility shift assay (EMSA). A representative EMSA gel is presented here (**b**) TAK1^f/f^ and TAK1^scko^ cells isolated from non-tamoxifen-treated mice were transfected with pNF-κB-Luc plasmid along with pRL-TK plasmid in 1:10 ratio for 24 h followed by treatment with 4-hydoxytamoxifen (TAM) for 48 h. The cells were then treated with 10 ng ml^−1^ TNFα for 18 h followed by measuring the amounts of luciferase and renilla activity in cell extracts using Dual luciferase assay kit. The fold change in luciferase/renilla ratio is presented here. *N*=3 in each group. *P<0.01, values significantly different from corresponding control TAK1^f/f^ or TAK1^scko^ cultures. #*P*<0.05, values significantly different from TNFα-treated TAK1^scko^ cultures. $*P*<0.05, values significantly different from control TAK1^f/f^ cultures. (**c**) Representative immunoblots presented here demonstrate the levels of p-IκBα, total IκBα, p100, p52, Bcl-2, cIAP1 and an unrelated protein GAPDH in TAK1^f/f^ and TAK1^scko^ cultures. (**d**) Densitometry analysis of ratio of phosphorylated and total IκBα protein in TAK1^f/f^ and TAK1^scko^ cultures. (**e**) Relative amounts of p100, p52, Bcl-2 and cIAP1 in TAK1^f/f^ and TAK1^scko^ cultures. TAK1^f/f^ and TAK1^scko^ cells isolated from non-tamoxifen-treated mice were transfected with vector alone or caIKKβ followed by treatment with 4-hydoxytamoxifen (TAM) for 48 h. The cells were then washed and incubated in growth medium and analysed at specific time points. Percentage of (**f**) apoptotic (AnnexinV^+^/PI^+^) (**g**) necroptotic (that is, PI^+^) cells in vector alone and caIKKβ-transfected TAK1^f/f^ and TAK1^scko^ cultures measured after 72 h of removal of TAM. Percentage of (**h**) cells in G2/M phase of cell cycle, (**i**) Pax7^+^/EdU^+^ cells, (**j**) Pax7^+^/MyoD^+^, (**k**) Pax7^−^/MyoD^+^ and (**l**) CM-H2DCFDA^+^ cells in vector alone or caIKKβ-transfected TAK1^f/f^ and TAK1^scko^ cultures measured after 48 or 72 h of removal of TAM. *N*=4 in each group for all the experiments in this figure. Error bars represent s.d. *P<0.01, values significantly different from corresponding TAK1^f/f^ or TAK1^scko^ cultures transfected with vector alone by paired *t*-test. #*P*<0.05, values significantly different from TAK1^scko^ cultures transfected with vector alone by paired *t*-test. $*P*<0.05, values significantly different from TAK1^f/f^ cultures transfected with vector alone by paired *t*-test.

TAK1 is an important upstream signalling molecule that mediates the activation of NF-κB and MAPK pathways. We first investigated the effects of inactivation of TAK1 on NF-κB pathway. Satellite cells isolated from hind limb muscle of non-tamoxifen-treated TAK1^f/f^ and TAK1^scko^ mice were treated with TAM for 48 h to induce Cre-mediated recombination. The cells were then washed to remove TAM and cultured for additional 36 h. As a stimulus to induce NF-κB, the cells were treated with TNF-α for different time intervals and the nuclear extracts made were analysed for DNA-binding activity of NF-κB by performing electrophoretic mobility shift assay. As shown in Fig. 8a, the basal level of NF-κB activity was found to be considerably reduced in TAK1^scko^ cultures compared with TAK1^f/f^ cultures. While TNF-α drastically increased the DNA-binding activity of NF-κB in TAK1^f/f^ cells, there was only a modest increase in TAK1^scko^ cells upon treatment with TNFα (Fig. 8a). Our results also showed that basal level of NF-κB reporter gene activity was significantly reduced in TAK1^scko^ cultures compared with TAK1^f/f^ cultures. Moreover, the TNFα-induced activation of NF-κB reporter gene was significantly inhibited in TAK1^scko^ cells compared with TAK1^f/f^ cells (Fig. 8b).

NF-κB can be activated through canonical or non-canonical pathways. In canonical pathway, IKKβ phosphorylates IκB leading to its proteolytic degradation and which allows nuclear translocation of p50/p65 complex. In non-canonical pathway, IKKα phosphorylates p100 subunit leading to its proteolytic processing into p52 subunit. Although canonical and non-canonical pathways are activated through distinct mechanisms, these pathways cross-talk^55^. By performing western blotting, we compared the levels of phosphorylated IκBα and p100/p52 proteins in TAK1^f/f^ and TAK1^scko^ cells. This analysis showed that the ratio of phosphorylated vs total IκBα protein was significantly reduced in TAK1^f/f^ cultures compared with TAK1^scko^ cultures (Fig. 8c,d). Intriguingly, we also found that the levels of both p100 and p52 proteins were significantly reduced in TAK1^scko^ cultures. Furthermore, the levels of NF-κB regulated pro-survival proteins such as Bcl-2 and cellular inhibitor of apoptosis (cIAP1) were significantly reduced in TAK1^scko^ cultures compared with TAK1^f/f^ cultures (Fig. 8c,e).

Since NF-κB is a pivotal regulator of cellular proliferation and survival^55^, we next investigated the effects of forced activation of NF-κB in TAK1-deficient satellite cells. TAK1^f/f^ and TAK1^scko^ cells were transfected with vector alone or a constitutively activate (ca) mutant of IKKβ. Interestingly, overexpression of ca IKKβ inhibited both early and late apoptosis in TAK1^scko^ cells (Fig. 8f). Overexpression of caIKKβ also significantly reduced the number of PI^+^ (necroptotic) cells in TAK1^scko^ cultures (Fig. 8g). Surprisingly, we found that overexpression of caIKKβ significantly increased the number of PI^+^ cells in TAK1^f/f^ cultures, suggesting that spurious activation of NF-κB can lead to cell death through necroptosis in WT satellite cells (Fig. 8g). Indeed, NF-κB is a well-known regulator of both cell survival and death^55^. Our results also showed that the forced expression of caIKKβ enhanced the number of cells in G2/M phase of cell cycle and increased the proliferation of Pax7^+^ cells in TAK1^scko^ cultures (Fig. 8h,i). We also observed a significant increase in the proportion of Pax7^+^/MyoD^+^ cells with concomitant decrease in proportion of Pax7^−^/MyoD^+^ cells in TAK1^scko^ cultures transfected with caIKKβ (Fig. 8j,k). Overexpression of caIKKβ significantly reduced ROS levels in TAK1^scko^ cultures transfected with caIKKβ. However, we found that caIKKβ increased oxidative stress in TAK1^f/f^ cells (Fig. 8l). It is possible that continued activation of IKKβ for longer duration increases oxidative stress that leads to satellite cell death through necroptosis. Collectively, these results suggest that TAK1 promotes satellite cell proliferation and survival through the activation of NF-κB transcription factor.

### TAK1 mediates the activation of JNK1/2 in satellite cells

**Figure 9:**
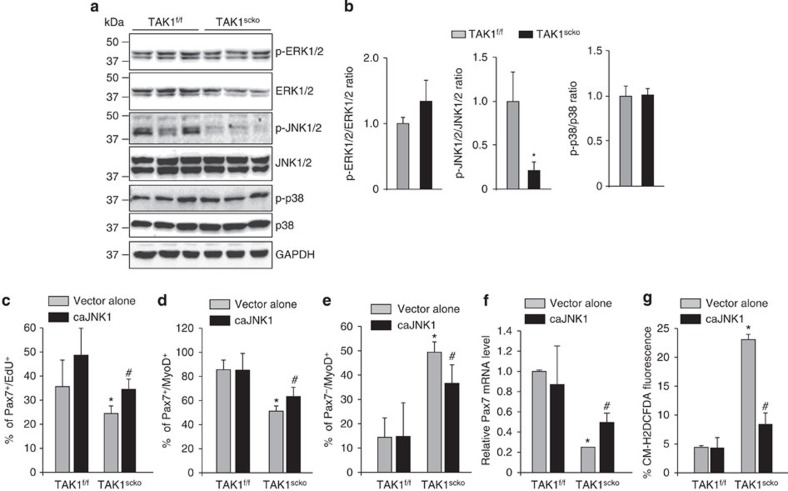
TAK1 mediates the activation of JNK1/2 in satellite cells. (**a**) Immunoblots presented here demonstrate the levels of phosphorylated and total ERK1/2, JNK1/2 and p38 MAPK protein in TAK1^f/f^ and TAK1^scko^ cultures. (**b**) Densitometry analysis of ratio of phosphorylated and total ERK1/2, JNK1/2 and p38 MAPK protein in TAK1^f/f^ and TAK1^scko^ cultures. TAK1^f/f^ and TAK1^scko^ cells isolated from non-tamoxifen-treated mice were transfected with vector alone or caJNK1 followed by treatment with 4-hydoxytamoxifen (TAM) for 48 h. The cells were then washed and incubated in growth medium for 48 h. Percentage of (**c**) Pax7^+^/EdU^+^ cells, (**d**) Pax7^+^/MyoD^+^ and (**e**) Pax7^−^/MyoD^+^ cells in vector alone or caJNK1-transfected TAK1^f/f^ and TAK1^scko^ cultures. (**f**) Relative mRNA levels of Pax7 in vector alone or caJNK1-transfected TAK1^f/f^ and TAK1^scko^ cultures. (**g**) Percentage of CM-H2DCFDA^+^ in TAK1^f/f^ and TAK1^scko^ cultures transfected with vector alone or caJNK1. *N*=4 in each group for all the experiments in this figure. Error bars represent s.d. *P<0.01, values significantly different from corresponding TAK1^f/f^ or TAK1^f/f^ cultures transfected with vector alone by unpaired or paired *t*-test. #*P*<0.05, values significantly different from TAK1^scko^ cultures transfected with vector alone by paired *t*-test.

We next investigated whether TAK1 affects the activation of MAPK in satellite cells. Although there was no effect on ERK1/2 and p38 MAPK, the levels of phosphorylated JNK1/2 were significantly reduced in TAK1^scko^ cultures compared with TAK1^f/f^ cultures (Fig. 9a,b). To understand the role of JNK signalling in survival and proliferation of TAK1-deficient cells, we studied the effect of forced expression of Flag MKK7B2Jnk1a1 (henceforth caJNK1) protein that functions as a constitutively active mutant of JNK1 (ref. 56). Overexpression of caJNK1 did not affect early or late apoptosis and necroptosis in TAK1^scko^ cells. However, we found a significant increase in the number of Pax7^+^/Edu^+^ cells in the TAK1^scko^ cultures upon overexpression of caJNK1 (Fig. 9c). Furthermore, a significant increase in the proportion of Pax7^+^/MyoD^+^ cells with concomitant decrease in the proportion of Pax7^−^/MyoD^+^ cells was noticeable upon overexpression of caJNK1 in TAK1^scko^ cultures (Fig. 9d,e). Overexpression of caJNK1 significantly increased the mRNA levels of Pax7 in TAK1^scko^ myogenic cultures (Fig. 9f). Finally, we observed that overexpression of caJNK1 significantly reduced the levels of ROS in TAK1^scko^ cells (Fig. 9g). These results suggest that TAK1 mediates satellite cell self-renewal and proliferation, at least in part, through the activation of JNK.

## Discussion

Satellite cells are primarily a quiescent cell population in adult skeletal muscle. However, like other stem cells, satellite cells also self-renew to maintain a pool of healthy cells^2^. In response to exercise, traumatic muscle injury or degenerative muscle disorder, these cells undergo several rounds of cell division to produce fusion competent myoblasts to support growth, repair and regeneration^1^,^3^,^5^. Although replenishment of satellite cell population is pivotal for maintaining skeletal muscle regenerative capacity^2^, a reduction in the pool or function of satellite cells can compromise muscle regenerative capacity and may eventually lead to muscle wasting similar to that observed during aging^57^ and in various genetic muscle disorders such as muscular dystrophy^3^,^58^,^59^. The results of the present study demonstrate that TAK1 is activated in satellite cells and plays an important role in survival, proliferation and maintenance of satellite cell pool and repair of skeletal muscle upon injury in adult mice.

Inactivation of TAK1 in satellite cells impairs early muscle regenerative program leading to severe deficit in repair of injured myofibers (Fig. 2). While there was a drastic reduction in the Pax7^+^ satellite cells in injured myofibers, the number of satellite cells was also found to be significantly reduced in naive skeletal muscle or on freshly isolated myofibers of TAK1^scko^ mice (Fig. 3). Our results further demonstrate that TAK1 is essential for the proliferation and survival of satellite cells. A marked reduction in the number of EdU^+^ nuclei was clearly evident in the regenerating myofibers of TAK1^scko^ mice compared with TAK1^f/f^ mice (Fig. 4a–c). Reduced proliferation of satellite cells was also evident on cultured single myofibers of TAK1^scko^ mice (Supplementary Fig. 3) and in purified TAK1^scko^ satellite cell cultures (Fig. 4d–g). Furthermore, pharmacological inhibition of TAK1 drastically reduced the proliferation of satellite cells (Supplementary Fig. 4). Interestingly, we also observed that the continued inhibition of TAK1 increases oxidative stress and causes cell death through induction of both apoptosis and necroptosis. (Fig. 6). These results are in agreement with other published reports also suggesting that TAK1 is essential for cellular homeostasis in other organs^22^,^35^,^36^,^37^,^41^. Reduced number of Pax7^+^ cells in naive and injured muscle of TAK1^scko^ mice with concomitant inhibition in the early markers of muscle regeneration are potentially due to diminished sustainability and increased mortality of satellite cells, which in turn leads to a depletion in the pool of satellite cells that facilitate the regenerative program upon injury.

A recent study has shown that Pax7 is also expressed in a rare subpopulation of spermatogonial stem cells^60^ and therefore there is a possibility that the tamoxifen-treatment protocol used in our study may also reduce the levels of TAK1 in spermatogonia leading to potential non-cell autonomous effects. However, we believe that this is not the case because ablation of TAK1 in cultured satellite cells leads to similar phenotypic outcomes as observed in skeletal muscle tissues of TAK1^scko^ mice. Furthermore, for our studies, we gave localized injections of BaCl_2_ to TA muscle only which should not affect the proliferation of Pax7^+^ cells in spermatogonia.

Pax7 is a pivotal regulator for satellite cell biogenesis, survival, specification and self-renewal^2^,^3^,^4^. Although exact mechanisms remain unknown, our results suggest that TAK1 is required for maintaining the expression of Pax7 in satellite cells (Fig. 5). While TAK1 appears to be required for the gene expression of Pax7, it has no role in regulating the gene expression of MyoD in proliferating satellite cells. Inhibition of TAK1 reduces the mRNA level of Pax7 but not MyoD (Fig. 5i–k). Increased levels of MyoD protein observed after inhibition of TAK1 may be a result of increased stability of MyoD protein. Indeed, previous studies have shown that the activation of NF-κB (a downstream target of TAK1) reduces the stability of MyoD in response to inflammatory cytokines^16^. Consistently, our results demonstrate that the inactivation of TAK1 inhibits basal levels, as well as TNFα-induced NF-κB activation in satellite cells (Fig. 8).

Previous studies have shown that TAK1 is required for the differentiation of myoblasts into myotubes^43^,^44^. Satellite cells first differentiate into myoblasts, which in turn fuse with injured myofibers to complete the repair process. Although we found reciprocal changes in the levels of Pax7 and MyoD, it is noteworthy that Pax7^−^/MyoD^+^ TAK1^scko^ cells may not undergo terminal differentiation because TAK1 is also required for the activation of p38 MAPK in myogenic lineage committed myoblasts to progress through differentiation^43^,^44^,^61^. Therefore, reduced differentiation potential of surviving TAK1-deficient myoblasts could be another reason for the deficit in muscle regeneration in TAK1^scko^ mice.

Accumulating evidence suggests that disruption of TAK1-mediated signalling can cause cell death through apoptosis or necroptosis^34^. Apoptosis has been traditionally considered as the physiological mode of cell death. However, necroptosis, which is morphologically related to necrosis, is now recognized as another mode of programmed cell death^39^. RIPK1 is activated through oligomerization of upstream adaptor molecules such as FADD and TNF receptor-associated death domain that are triggered by TNFα or Fas ligand. Subsequently, activated RIPK1 activates RIPK3 resulting in necroptosis^38^,^39^. Inactivation of TAK1 induces oxidative stress in keratinocytes leading to their reduced survival^52^. Intriguingly, oxidative stress has been found to induce necroptosis in some cell types^53^. Our results demonstrate that the inhibition of TAK1 significantly increases ROS accumulation in satellite cells (Fig. 6k,l). Furthermore, we have found that while only a small percentage of TAK1^scko^ satellite cells undergo apoptosis, a vast majority of them die through necroptosis (Fig. 7). Although there are reports suggesting that satellite cells undergo apoptotic cell death^62^,^63^,^64^, our study provides initial evidence that necroptosis could be another mechanism of satellite cell mortality especially in the conditions where TAK1-mediated signalling is disrupted.

NF-κB is one of the important signalling pathways activated through TAK1-dependent mechanisms. Activation of TAK1 complex leads to the phosphorylation of IKKβ in its activation domain leading to the activation of NF-κB through phosphorylation and proteolytic degradation of IκBα. Dissociation of IκB unmasks the nuclear localization signal of NF-κB, permitting nuclear import and transcriptional activity^15^,^16^,^55^. Constitutive activation of NF-κB inhibits terminal differentiation of myogenic cells in response to inflammatory cytokines and in genetic muscle disorders such as muscular dystrophy^15^,^16^. However, the role of NF-κB in satellite cell proliferation and survival remains poorly understood. Our results demonstrate that inactivation of TAK1 inhibits NF-κB pathways in satellite cells and this appears to be a critical mechanism for the poor survival and proliferation of TAK1-deficient satellite cells. Forced activation of NF-κB through overexpression of a constitutive active mutant of IKKβ improved survival and increased proliferation of TAK1^scko^ satellite cells. Moreover, overexpression of caIKKβ increased the proportion of Pax7^+^/MyoD^+^ cells in TAK1^scko^ cultures (Fig. 8). While the role of NF-κB in prevention of apoptosis is well known, we have found that overexpression of caIKKβ also prevents necroptotic cell death in TAK1-deficient cells (Fig. 8). These results are in agreement with a recently published report demonstrating that the inhibition of IKKβ can induce necroptosis in malignant B lymphocytes^65^. Furthermore, there is also a report suggesting that NF-κB is essential to prevent oxidative stress and necroptosis in response to interferon-γ (ref. 66). Although we observed improvement in the proliferation and survival of TAK1^scko^ satellite cells upon overexpression of caIKKβ, it is notable that spurious activation of NF-κB by itself can cause satellite cell death. Overexpression of caIKKβ significantly increased oxidative stress and cell death by necroptosis in WT (that is, TAK1^f/f^) cells (Fig. 8g,l), which is consistent with previous reports that NF-κB regulates both cell survival and death^55^.

We have found that the phosphorylation of JNK was also significantly reduced in the TAK1^scko^ cultures compared with TAK1^f/f^ satellite cells (Fig. 9a,b). JNK1 is an important regulator of cellular proliferation. Depending on the upstream stimuli and cell type, activation of JNK can promote cellular viability or apoptosis^67^. A recent study has shown that the activation of JNK through disruption of MEK-5 increases the proportion of Pax7^+^ cells during regenerative myogenesis^11^. Our results demonstrate that while overexpression of constitutively active mutant of JNK1 had no effect on cell survival, it significantly increased the proportion of Pax7^+^ cells and mRNA levels of Pax7 in TAK1^scko^ cultures (Fig. 9c,e). Furthermore, caJNK1 significantly reduced oxidative stress in TAK1-deficient satellite cells (Fig. 9g). Therefore, inhibition of JNK appears to be another reason for the reduced proliferation of satellite cells upon inactivation of TAK1. While our experiments indicate that TAK1 regulates satellite cell function through activation of NF-κB and JNK1/2, a drawback of these experiments is that the overexpression of caIKKβ or caJNK1 can cause supra-physiological activation of NF-κB or JNK pathways, respectively. Furthermore, our rescue experiments are limited to cultured satellite cells. Future studies will determine whether forced activation of NF-κB or JNK1 in physiological range can promote satellite cell survival and function in TAK1-deficient mice.

In summary, our study provides initial evidence that TAK1 is required for satellite cell proliferation and survival and for regeneration of adult skeletal muscle. Future studies should determine whether the disruption of TAK1 is a potential cause for the depletion of satellite cells and their function in various muscle degenerative disorders and during aging.

## Methods

### Generation of satellite cell-specific TAK1 knockout mice

Satellite cell-specific TAK1 knockout mice (that is, TAK1^scko^) were generated by crossing Pax7^CreER^ mice (Jax Strain: B6;129-Pax7^tm2.1(cre/ERT2)Fan^/J) with floxed TAK1 (that is, TAK1^f/f^) mice^36^. All mice were in the C57BL6 background and their genotype was determined by PCR from tail DNA. Deletion of TAK1 in satellite cells of TAK1^scko^ mice was confirmed by PCR using the following primers set, which detect truncated form of Tak1 (Tak1Δ): 5′-CACCAGTGCTGGATTCTTTTTGAGGC-3′ and 5′-GGAACCCGTGGATAAGTGCACTTGAAT-3′. TNF receptor was used as a loading control and detected by PCR using the following primer set: 5′-TGTGAAAAGGGCACCTTTACGGC-3′ and 5′-GGCTGCAGTCCACGCACTGG-3′ as described^52^.

### Animals and skeletal muscle injury

Mice were housed in individual cages in an environmentally controlled room (23 °C, 12-h light–dark cycle) with *ad libitum* access to food and water. All experiments were performed on 8–10-week-old male (24–28 g) and female mice (21–25 g); mice of the same sex and age were used for each individual experiment. For inducible inactivation of TAK1, TAK1^f/f^ and TAK1^scko^ mice were injected intraperitoneally with tamoxifen (10 mg Kg^−1^ body weight) for 4 consecutive days and kept on tamoxifen-containing standard chow (Harlan Laboratories, Madison, WI) for the entire duration of the experiment. For muscle injury experiments, 100 μl of 1.2% BaCl_2_ (Sigma Chemical Co.) dissolved in saline was injected into the TA muscle of mice. For one experiment, mice were given intraperitoneal injection of EdU (100 μg per mouse) at day 3 or day 5 after intramuscular injection of 1.2% BaCl_2_. At various time points, TA muscle was collected from euthanized mice for biochemical and histology studies. All experimental protocols with mice were in accordance with approved ethical guidelines of the Institutional Animal Care and Use Committee at the University of Louisville.

### Histology and morphometric analysis

Hind limb muscle from mice were isolated and frozen in liquid nitrogen and sectioned in a microtome cryostat. For the assessment of muscle morphology, 10-μm thick transverse sections of TA muscle were stained with H&E. The sections were examined under Nikon Eclipse TE 2000-U microscope (Nikon). For quantitative analysis, CSA of myofibers was analysed in H&E-stained TA muscle sections using Nikon NIS Elements BR 3.00 software (Nikon). For each muscle, the distribution of fibre CSA was calculated by analysing ∼200 myofibers.

### Isolation, culture and staining of single myofibers

For single-myofiber isolation, EDL muscle was dissected tendon to tendon from mice and enzymatically digested in DMEM medium supplemented with collagenase (400 IU ml^−1^, Worthington) at 37 °C for 45 min. Post digestion, single myofibers were released by trituration in myofiber culture medium (DMEM with 10% fetal bovine serum (FBS)). Suspended fibres were cultured in 60-mm horse serum-coated plates in DMEM supplemented with 10% FBS (Invitrogen), 2% chicken embryo extract (Accurate Chemical, Westbury, NY; Peprotech, Rocky Hill, NJ) and 1% penicillin–streptomycin for 3 days. Freshly isolated fibres were then fixed in 4% PFA and stained for Pax7 and DAPI.

### Satellite cell cultures

Mice were killed and TA and gastrocnemius muscles were isolated and excess connective tissues and fat were cleaned in sterile PBS. Muscle tissues were then minced into coarse slurry and enzymatically digested at 37 °C for 1 h by adding 400 IU ml^−1^ collagenase II (Worthington). The digested slurry was spun, pelleted and triturated several times and then passed through a 70-μm and then 30-μm cell strainer (BD Falcon). The filtrate was spun at 1,000*g* and suspended in myoblast growth medium (Ham's F-10 medium with 20% FBS supplemented with 10 ng ml^−1^ of basic fibroblast growth factor). Cells were first refed after 3 days of initial plating. For the first few passages, cells were also enriched by preplating for selection of pure myoblast population. Upon selection, the cells were cultured in a 1:1 ratio of myoblast growth medium and growth medium (DMEM with 20% FBS). TAK1 in cultured TAK1^scko^ myogenic cells was deleted by incubation in 10 ng ml^−1^ TAM (Sigma Chemical Co.) for 48 h.

### Immunofluorescence

For immunohistochemistry study, frozen TA muscle sections or 3.7% PFA fixed single myofibers or cultured myogenic cells were blocked in 1% bovine serum albumin in PBS for 1 h and incubated with anti-Pax7 (1:10, Developmental Studies Hybridoma Bank, DSHB, University of Iowa, Iowa City, IA), anti-MyoD (1:200, Santa Cruz Biotechnology), anti-E-MyHC (1:200, DSHB, University of Iowa, Iowa City, IA), anti-MF20 (1:250, DSHB, University of Iowa, Iowa City, IA), anti-laminin (1:100, Sigma Chemical Company) in blocking solution at 4 °C overnight under humidified conditions. The sections were washed briefly with PBS before incubating with Alexa Fluor 488- or 546-conjugated secondary antibody (1:3,000, Invitrogen) for 1 h at room temperature and then washed three times for 5 min with PBS. The slides were mounted using fluorescence medium (Vector Laboratories) and visualized at room temperature on Nikon Eclipse TE 2000-U microscope (Nikon), a digital camera (Nikon Digital Sight DS-Fi1), and Nikon NIS Elements BR 3.00 software (Nikon). Image levels were equally adjusted using Adobe Photoshop CS6 software (Adobe).

### Cell proliferation assay

Satellite cell proliferation was assayed by labelling the cells with EdU for 90 min using Click-iT EdU Cell Proliferation Assay kit (Invitrogen). Nuclei were counterstained with DAPI for 30 min at room temperature. Images were visualized on Nikon Eclipse TE 2000-U microscope (Nikon), a digital camera (Nikon Digital Sight DS-Fi1), and analysed using Nikon NIS Elements BR 3.00 software (Nikon).

### Short hairpin RNA TAK1 and plasmid constructs

The pLKO.1-mCherry-Puro plasmid was kindly provided by Dr Renzhi Han of the Ohio State University. The target short interfering RNA sequences was identified using BLOCK-iT RNAi Designer online software (Life Technologies). At least two short interfering RNA sequences were tested for efficient knockdown of target TAK1 mRNA. The shRNA oligonucleotides were synthesized to contain the sense strand of target sequences for mouse TAK1 (that is, 5′-GGTGCTGAACCATTGCCTTAC-3′), short spacer (CTCGAG) and the reverse complement sequences followed by five thymidines as an RNA polymerase III transcriptional stop signal. Oligonucleotides were annealed and cloned into pLKO.1-mCherry-Puro with AgeI/EcoRI sites. The insertion of shRNA and complementary (cDNA) sequence in the plasmids was confirmed by DNA sequencing. Constitutive active mutant of IKKβ (that is, IKK-2 S177E S181E) was a gift from Professor Anjana Rao (Addgene plasmid # 11105). pCDNA3 Flag MKK7B2Jnk1a1 was a gift from Roger Davis (Addgene plasmid # 19726). pNF-κB-Luc plasmid was purchased from Clontech. pRL-TK plasmid was from Promega (Madison, WI).

### Gene transfer by electroporation

To overexpress specific cDNA or shRNA in primary satellite cells, plasmid DNA was introduced into cells by electroporation (1,500 V, 10 ms for duration, three pulses) using the Neon transfection system following a protocol suggested by the manufacturer (Invitrogen).

### Transient transfection and reporter gene activity

TAK1^f/f^ and TAK1^scko^ cells plated in six-well tissue culture plates were transfected with pNF-κB-Luc plasmid using Lipofectamine transfection reagent according to the manufacturer's protocol (Invitrogen). Transfection efficiency was controlled by cotransfection of cells with Renilla luciferase-encoding plasmid pRL-TK (Promega). The cells were then treated with 10 ng ml^−1^ recombinant TNFα (R&D Systems) for 18 h. The specimens were processed for luciferase expression using Dual-Luciferase assay systems with reporter lysis buffer according to the manufacturer's instructions (Promega). Luciferase measurements were made using a luminometer (Berthold Detection Systems).

### Lactate dehydrogenase assay

The amount of LDH in culture supernatants was measured using a LDH Cytotoxicity Assay kit and following the protocol suggested by the manufacturer (Thermo Scientific).

### Electrophoretic mobility shift assay

DNA-binding activity of NF-κB was measured by performing electrophoretic mobility shift assay. In brief, 15 μg of nuclear extracts prepared from TNF-treated or untreated TAK1^f/f^ or TAK1^scko^ cells was incubated with 16 fmol of 32P end-labelled NF-κB consensus oligonucleotide 5′-AGTTGAGGGGACTTTCCCAGGC-3′ (Promega) at 37 °C for 20 min, and the DNA–protein complex was resolved on a 7.5% native polyacrylamide gel. The radioactive bands from the dried gel were visualized and quantitated by PhosphorImager (Molecular Dynamics, Sunnyvale, CA) using ImageQuaNT software.

### Immunoprecipitation and western blot

Quantitative estimation of various proteins was done by performing western blot. TA muscle of mice or cultured myogenic cells were washed with PBS and homogenized in lysis buffer (50 mM Tris-Cl (pH 8.0), 200 mM NaCl, 50 mM NaF, 1 mM dithiotheritol, 1 mM sodium orthovanadate, 0.3% IGEPAL and protease inhibitors). Approximately, 100 μg of protein was resolved on each lane on 10% SDS–polyacrylamide gel electrophoresis, electrotransferred onto nitrocellulose membrane, probed using specific antibody at indicated dilution (Supplementary Table 1) and detected by chemiluminescence. For one experiments, cell extracts were immunoprecipitated using 1.5 μg of anti-FADD at 4 °C for overnight. The antibody–protein complex was collected using Protein A/G Sepharose beads and washed with lysis buffer. The proteins were then run on 10% SDS–polyacrylamide gel electrophoresis gel and immunoblotted with anti-RIP1 or anti-FADD. Bound antibodies were detected by secondary antibodies conjugated to horseradish peroxidase (Cell Signaling Technology). Signal detection was performed by an enhanced chemiluminescence detection reagent (Bio-Rad). Approximate molecular masses were determined by comparison with the migration of prestained protein standards (Bio-Rad). Quantitative estimation of the bands' intensity was performed using ImageJ software (National Institute of Health, Bethesda, MD). Uncropped blots are shown in Supplementary Fig. 6.

### Flow cytometry

For the detection of phospho-TAK1 in satellite cells, single-cell suspension prepared from uninjured and injured hind limb muscle WT mice were incubated with antibodies against CD45, CD31, Sca1 and Ter119 for negative selection (all PE conjugated, eBiosciences), and with α7-integrin (MBL International) and Alexa Fluor 488 for positive selection. The cells were then fixed with 1% PFA and permeabilized using 0.2% Triton X-100. The cells were then incubated with anti-phospho-TAK1 (Thr184/187) and Alexa 647 secondary antibody (Molecular Probes). FACS analysis was performed on a C6 Accuri cytometer (BD Biosciences) equipped with two lasers.

For cell cycle analysis, cells were collected and fixed in 70% ethanol overnight. The fixed cells were treated with RNase for 20 min stained with 5 μg ml^−1^ of PI for 30 min at 37 °C and analysed by FACS. For ROS detection, cells were collected and incubated with 10 ng ml^−1^ of CM-H2DCFDA (Invitrogen, Carlsbad, CA, USA) for 20 min and analysed by flow cytometry. Apoptosis was assessed by Annexin V/PI staining, whereas necroptosis was detected by PI staining alone followed by FACS according to the manufacturer's instructions (BD Biosciences).

### RNA isolation and quantitative real-time PCR

We used RNeasy Mini Kit (Qiagen) to extract RNA from cultured myogenic cells or skeletal muscle tissues of mice. Any contaminating DNA was removed using the DNA-free kit from Ambion (Austin, TX). Quantity of RNA was analysed using NanoDrop instrumentation (NanoDrop Technologies, Wilmington, DE). Purified RNA (1 μg) was used to synthesize first strand of cDNA by reverse transcription system using Ambion's oligo-dT primer and Qiagen's Omniscript reverse transcriptase according to the manufacturer's instructions. The first strand of cDNA reaction (0.5 μl) was subjected to real-time PCR amplification using gene-specific primers. The primers were designed using Vector NTI Xi software (Invitrogen). The sequence of the primers used in qRT-PCR assay is described in Supplementary Table 2.

Quantification of mRNA was done using the SYBR Green method on ABI Prism 7300 Sequence Detection System (Applied Biosystems, Foster City, CA). Approximately, 25 μl of reaction volume was used for the real-time PCR assay that consisted of 2 × (12.5 μl) Brilliant SYBR Green QPCR Master Mix (Applied Biosystems), 400 nM of primers (0.5 μl each from the stock), 11 μl of water and 0.5 μl of template. The thermal conditions consisted of an initial denaturation at 95 °C for 10 min followed by 40 cycles of denaturation at 95 °C for 15 s, annealing and extension at 60 °C for 1 minute and a final step melting curve of 95 °C for 15 s, 60 °C for 15 s and 95 °C for 15 s. All reactions were carried out in duplicate to reduce variation. The data were analysed using SDS software version 2.0, and the results were exported to Microsoft Excel for further analysis. Data normalization were accomplished using the endogenous control β-actin and the normalized values were subjected to a 2^−ΔΔCt^ formula to calculate the fold change between the control and experimental groups. The formula and its derivations were obtained from the ABI Prism 7900 Sequence Detection System user guide.

### Statistical analyses and general experimental design

We calculated sample size using size power analysis methods for *a priori* determination based on the s.d. and the effect size was previously obtained using the experimental procedures employed in the study. For animal studies, we estimated sample size from expected number of TAK1^scko^ mice and littermate TAK1^f/f^ controls. We calculated the minimal sample size for each group as eight animals. Considering a likely drop-off effect of 10%, we set sample size of each group of six mice. For some experiments, three to four animals were found sufficient to obtain statistical differences. Animals with same sex and same age were employed to minimize physiological variability and to reduce s.d. from mean. The exclusion criteria for animals were established in consultation with a veterinarian and experimental outcomes. In case of death, skin injury, sickness or weight loss of >10%, the animal was excluded from analysis. Tissue samples were excluded in cases such as freeze artefacts on histological section or failure in extraction of RNA or protein of suitable quality and quantity. Animals from different breeding cages were included by random allocation to the different experimental groups. Animal experiments were blinded using number codes till the final data analyses were performed. Statistical tests were used as described in the Figure legends. Results are expressed as mean±s.d. Statistical analyses used two-tailed Student's *t*-test to compare quantitative data populations with normal distribution and equal variance. A value of *P*<0.05 was considered statistically significant unless otherwise specified.

## Additional information

**How to cite this article:** Ogura, Y. *et al*. TAK1 modulates satellite stem cell homeostasis and skeletal muscle repair. *Nat. Commun.* 6:10123 doi: 10.1038/ncomms10123 (2015).

## Supplementary Material

Supplementary Information Supplementary Figures 1-6 and Supplementary Tables 1-2.
