# Supplementary material for: TAK1 modulates satellite stem cell homeostasis and skeletal muscle repair
Source: Nat Commun. 2015 Dec 9;6:10123. doi: 10.1038/ncomms10123 (PMC4682113; doi:10.1038/ncomms10123)
Supplement: Supplementary Information — Supplementary Figures 1-6 and Supplementary Tables 1-2. [file ncomms10123-s1.pdf]

Supplementary FIGURE 1

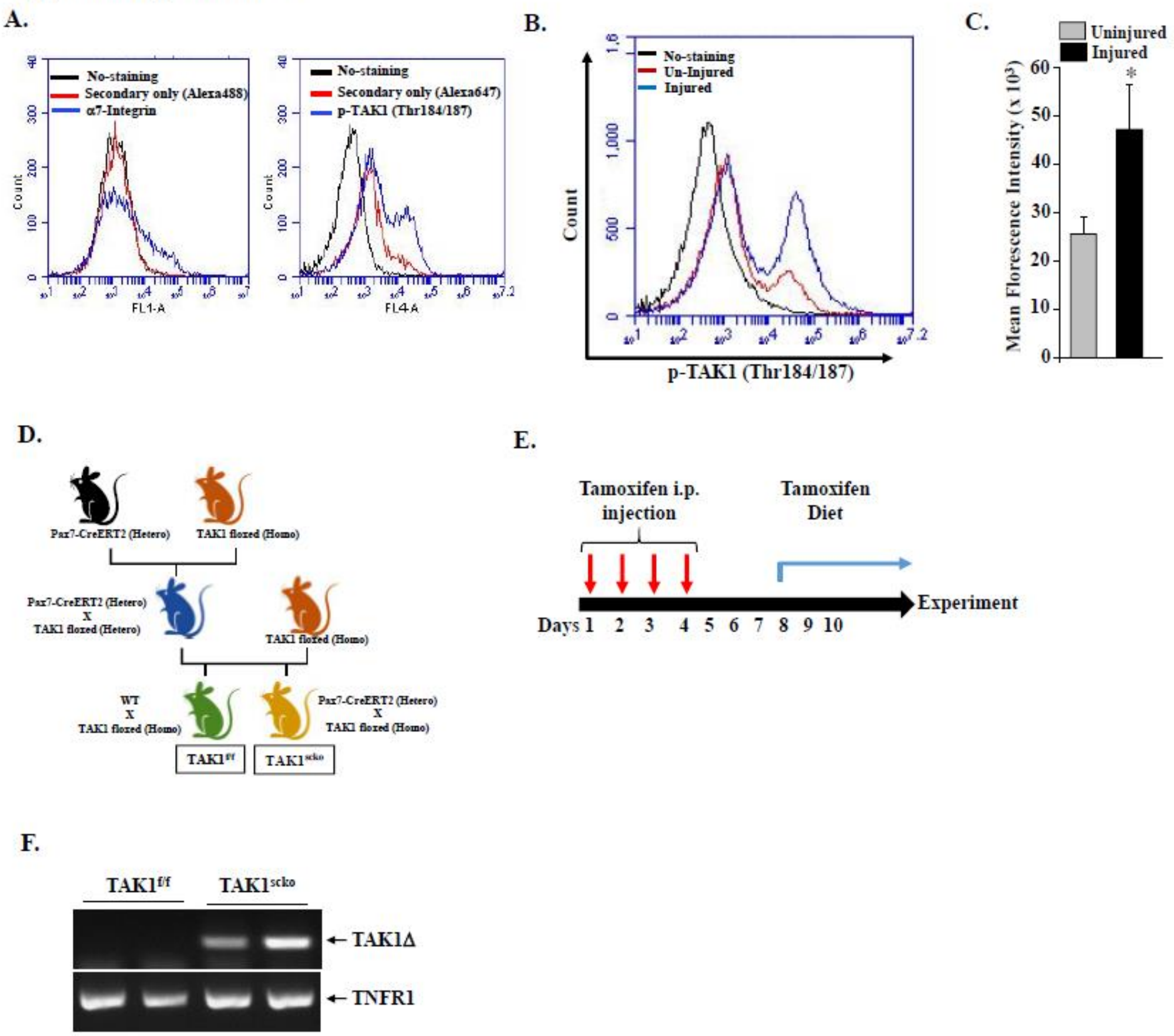

Supplementary Fig. 1: Detection of phosphorylated TAK1 in satellite cells and generation of TAK1$^{f/f}$ and TAK1^{scko} mice. (A) Representative histograms showing the specificity of α7-integrin and phospho-TAK1 antibodies in FACS analysis. **(B)** Representative histogram presented here demonstrate the levels of phospho-TAK1 (Thr184/187) in satellite cells of uninjured and 5d-injured TA muscle of wild-type mice. **(C)** Quantification florescence intensity in FACS analysis for phospho-TAK1 (Thr184/187) in satellite cells of uninjured and injured TA muscle of wild-type mice. N=3 in each group. Error bars represents standard deviation (SD). *p<0.01, values significantly different from contralateral uninjured TA muscle by unpaired t test. **(D)** Schematic illustration of the breeding strategy to obtain TAK1^{scko} and littermate TAK1$^{f/f}$ mice. **(E)** Treatment protocol for tamoxifen-induced Cre recombination in TAK1^{scko} mice. **(F)** PCR analysis of genomic DNA from 4-hydroxy tamoxifen (TAM) treated TAK1$^{f/f}$ and TAK1^{scko} cells using primer sets which detect truncated TAK1 and TNF receptor I (TNFR1). Representative PCR gel images presented here demonstrate that in vitro treatment with TAM for 48h efficiently delete kinase domain of TAK1 in TAK1^{scko} cells but not in TAK1$^{f/f}$ cells.

Supplementary FIGURE 2

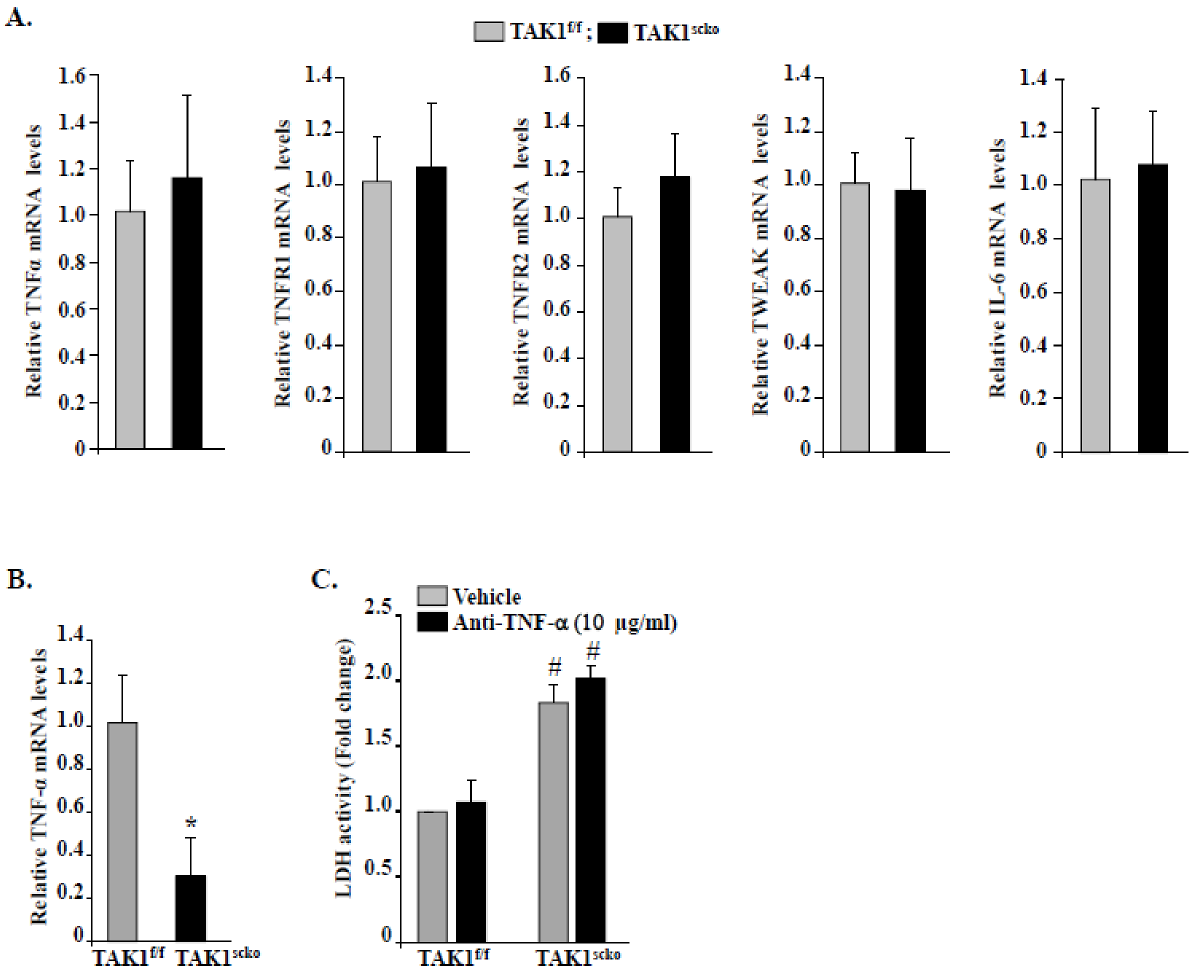

Supplementary Fig 2: Effect of ablation of TAK1 in satellite cells on the gene expression of inflammatory cytokines. (A) Relative mRNA levels of TNF-α, TNF receptor (TNFR) 1, TNFR2, TWEAK, and IL-6 in 5d-injured TA muscle of $TAK1^{f/f}$ and $TAK1^{scko}$ mice measured by QRT-PCR assays. **(B)** Relative mRNA levels of TNF-α in cultured $TAK1^{f/f}$ and $TAK1^{scko}$ cultures measured 72h after removal of TAM. **(C)** Primary myogenic cells isolated from non tamoxifen-treated $TAK1^{f/f}$ and $TAK1^{scko}$ mice were incubated with TAM for 48h. The cells were then washed and incubated in growth medium containing vehicle alone or mouse TNF-α neutralizing antibody (GeneTex, Clone # MAB0856) and after 72h the amounts of LDH in culture supernatants was measured. N=4 in each group for all the experiments. Error bars represent standard deviation (SD). *p<0.05, values significantly different from $TAK1^{f/f}$ cultures by unpaired t test. $^{\#}$p<0.05, values significantly different from corresponding $TAK1^{f/f}$ or $TAK1^{scko}$ cultures incubated with vehicle alone by paired t test.

Supplementary FIGURE 3

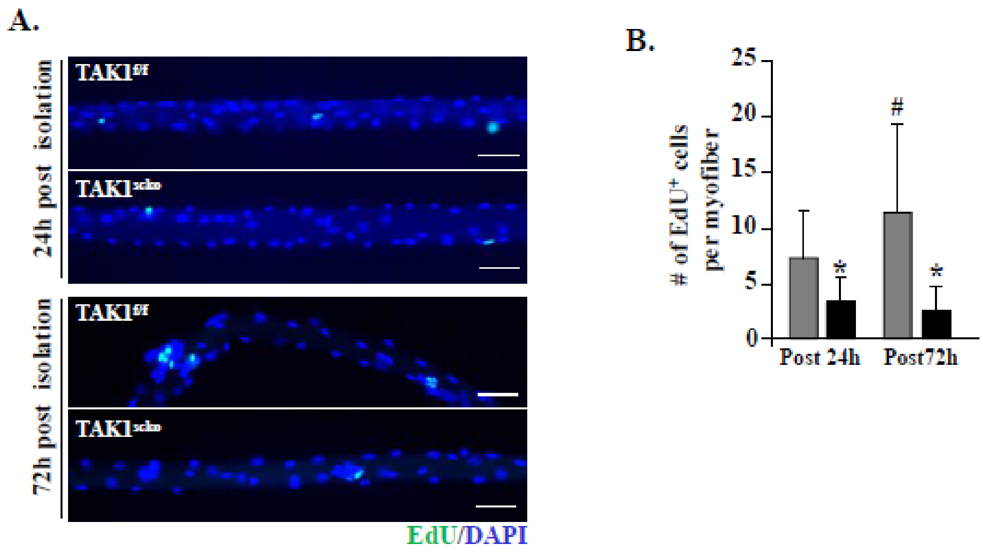

Supplementary Fig. 3: Role of TAK1 in proliferation of myofiber-associated satellite cells. Single myofibers cultures were established from EDL muscle of tamoxifen-treated TAK1$^{f/f}$ and TAK1^{scko} mice and cultured for 24h or 72h. For last 90 min, the cells were pulse labelled with EdU by addition of EdU in culture medium. The myofibers cultures were then fixed with paraformaldehyde and stained for EdU. Nuclei was labelled by incubation in DAPI solution. **(A)** Representative merged images of EdU and DAPI staining are presented here. Scale bar: 100µm. **(B)** Quantification of number of EdU^{+} nuclei per myofibers. N=3 mice per group. 10-12 myofiber were analyzed from each mouse. Error bars represent standard deviation (SD). *p<0.05, values significantly different from TAK1$^{f/f}$ cultures at 24h or 72h by paired t test. $^{\#}$p<0.05, Values significantly different from TAK1$^{f/f}$ cultures at 24h by paired t test.

Supplementary FIGURE 4

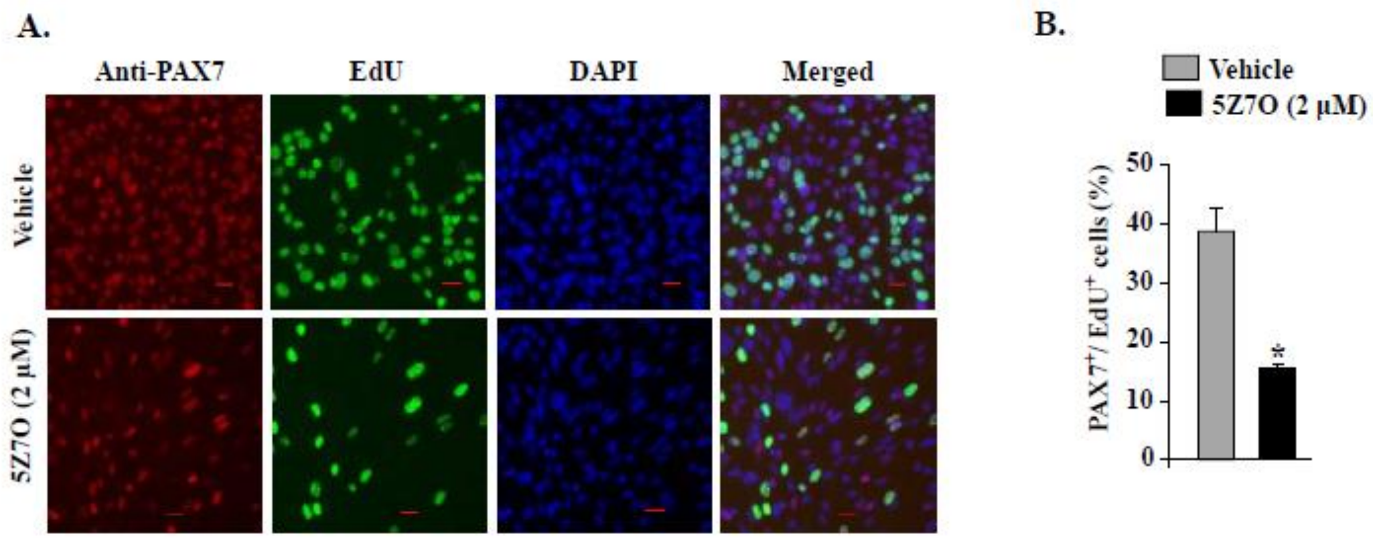

Supplementary Fig. 4: Effect of pharmacological inhibition of TAK1 on proliferation of satellite cells. Primary myogenic cells isolated from hind limb muscle of WT mice were treated with vehicle alone (i.e. DMSO) or 2 µM 5Z7O for 24h. For last 90 minutes, the cells were pulse labelled with EdU by addition of EdU in culture medium. The cultures were stained with anti-Pax7 and EdU. The nuclei were labeled using DAPI. **(A)** Representative individual and merged images of $TAK1^{f/f}$ and $TAK1^{scko}$ cultures after staining for Pax7, EdU, and nuclei (i.e. DAPI). Scale bar 20µm. **(B)** Quantitative estimation of percentage of $Pax7^{+}/EdU^{+}$ double positive cells in control and 5Z7O-treated cultures. N=4 in each group. Error bars represent standard deviation (SD). *p<0.01, values significantly different from vehicle treated cultures by unpaired t test.

Supplementary FIGURE 5

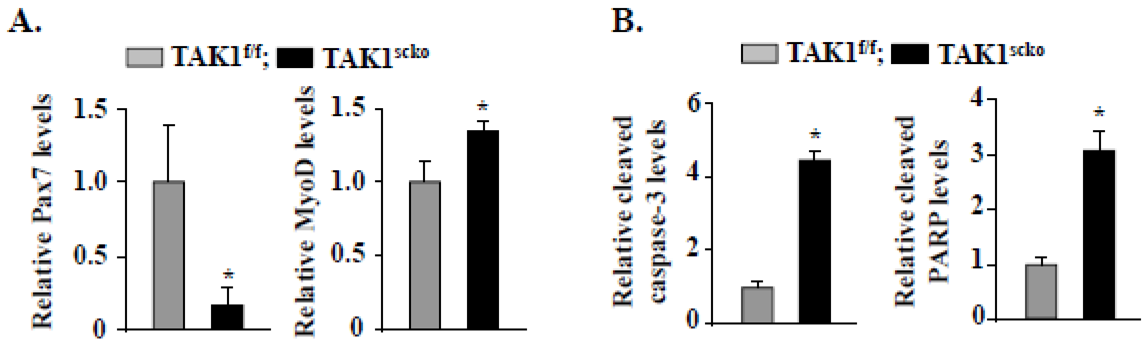

Supplementary Fig. 5: Densitometry quantitative estimation of bands in immunoblots performed for TAK1$^{f/f}$ and TAK1^{scko} cultures. Immunoblots for this analysis are presented in the main figures. Densitometry quantification of amounts of **(A)** Pax7 and MyoD protein (from Fig. 5D), **(B)** Cleaved caspase-3 and cleaved PARP (from Fig. 6D) in TAK1$^{f/f}$ and TAK1^{scko} cultures. N=3 in each group. Error bars represent standard deviation (SD). *p<0.05, values significantly different from corresponding TAK1$^{f/f}$ cultures by unpaired t test.

Supplementary FIGURE 6:

Fig. 1H

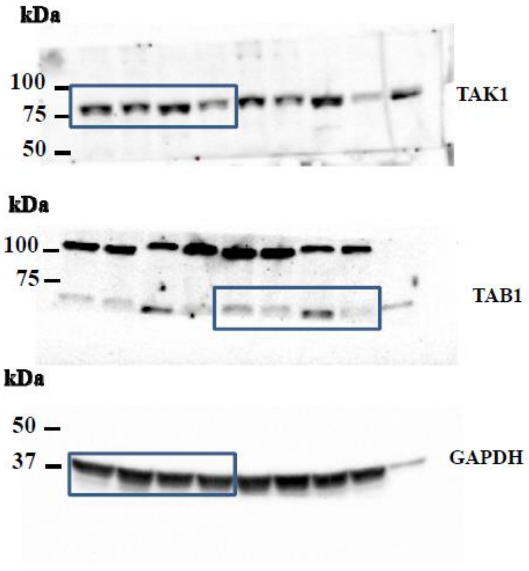

Fig. 2E

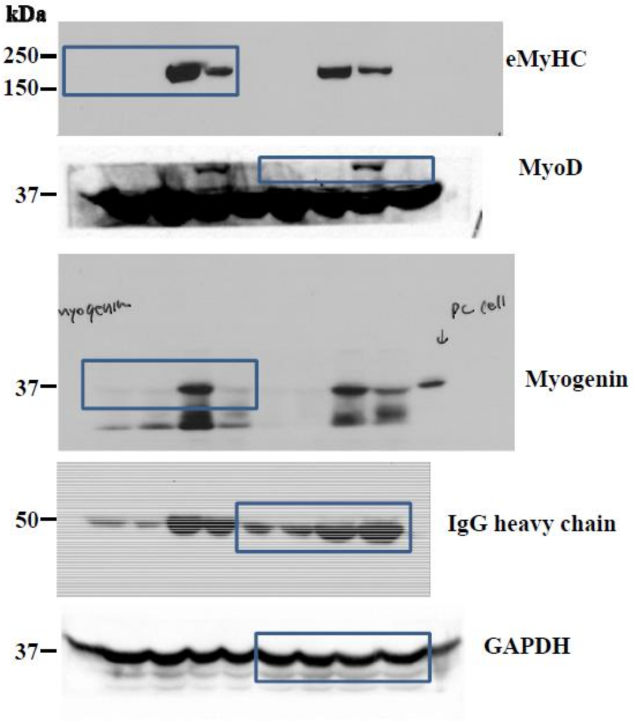

Fig. 4H

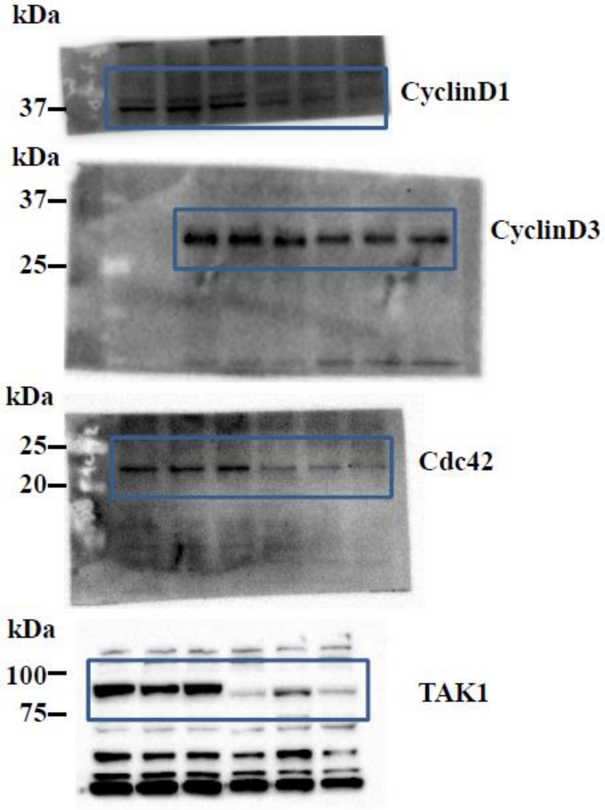

Supplementary FIGURE 6 (Continuation)

Fig. 5D

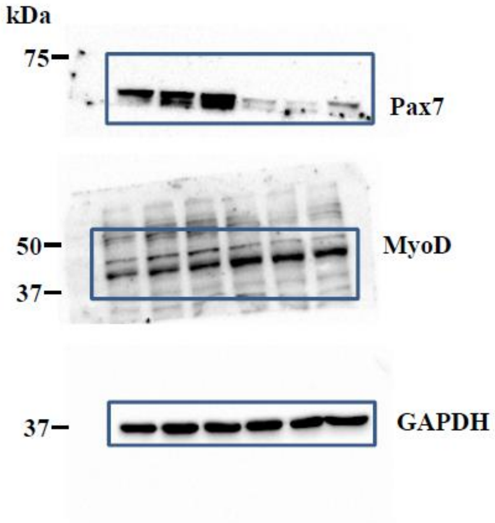

Fig. 5H

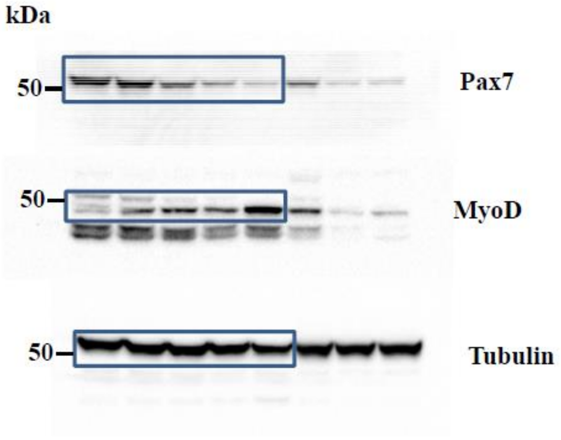

Fig. 6D

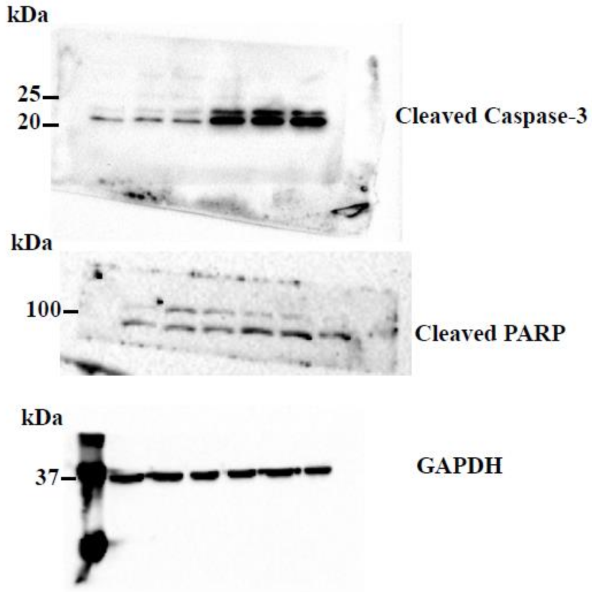

Fig. 6E

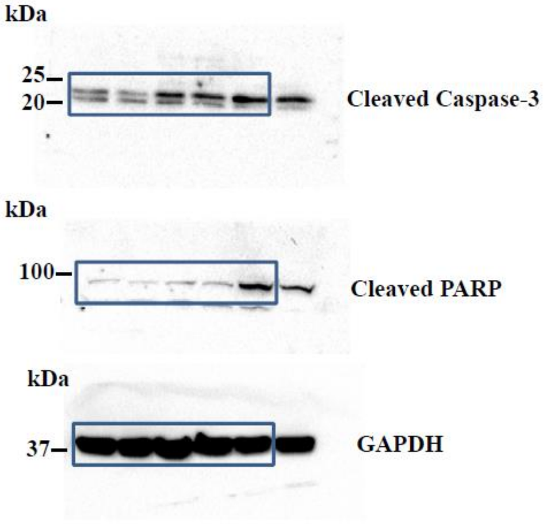

Supplementary FIGURE 6 (Continuation)

Fig. 6H

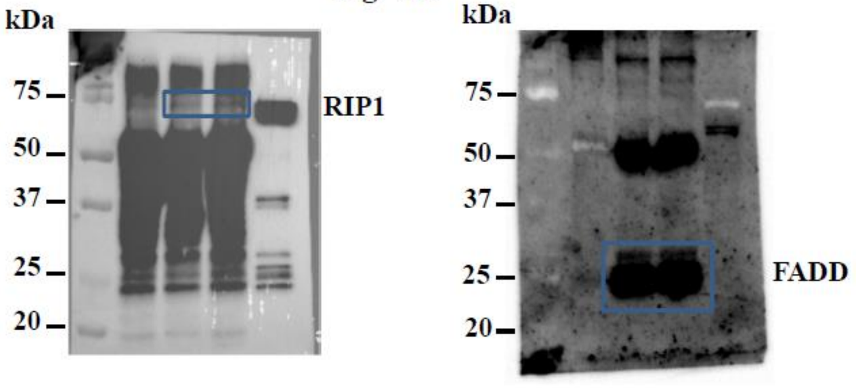

Fig. 6I

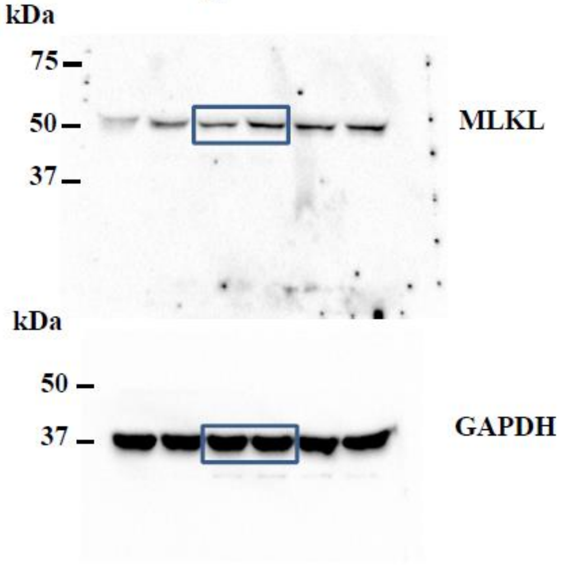

Supplementary FIGURE 6 (Continuation)

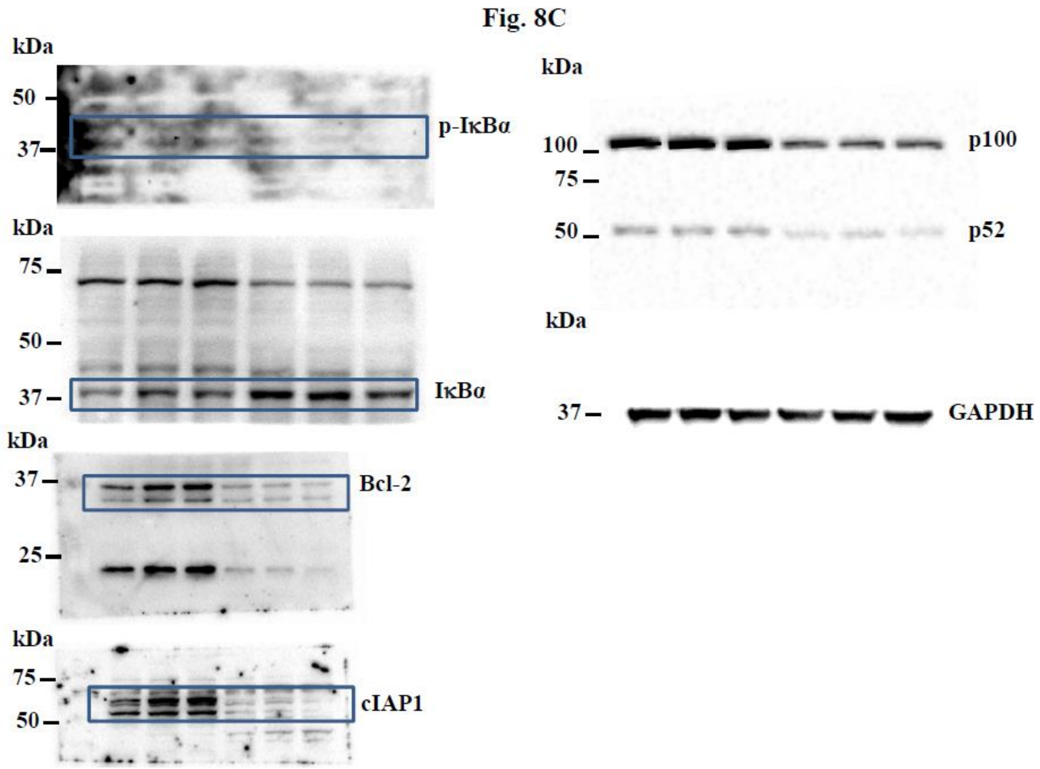

Supplementary FIGURE 6 (Continuation)

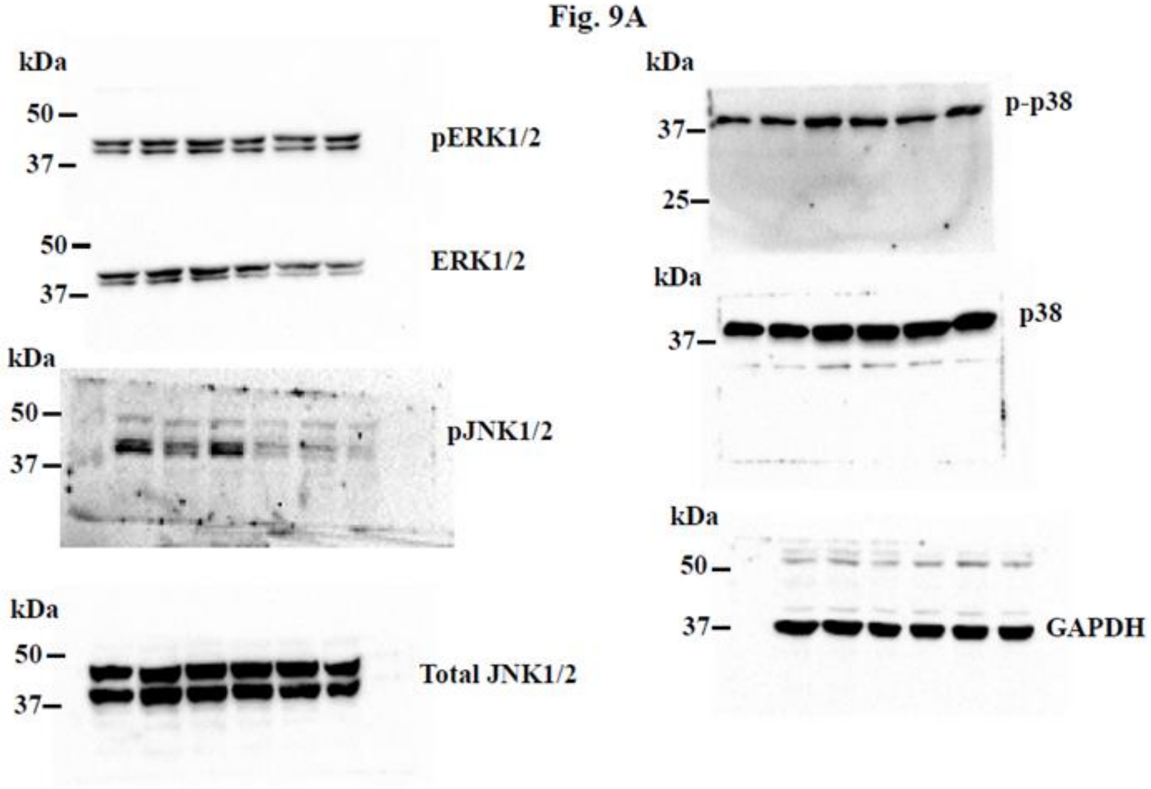

Supplementary Fig. 6. Uncropped images of immunoblots with molecular weight markers. The immunoblots presented here are the uncropped images for their corresponding figures in main manuscript. Bands marked in the rectangles were used where there are multiple bands in the same immunoblots. Molecular weight markers are presented on left side of these immunoblots.

Supplementary Table 1: Antibodies and their dilution used in this study.

Antibody	Company	Dilution	Analysis
Monoclonal rabbit-anti-phospho-TAK1	Thermo Scientific # MA5-15073	1:50	FACS
Monoclonal mouse-anti-Integrin α7	MBL International Corporation	1:240	FACS
Monoclonal mouse-anti-CD31	eBIOSCIENCE #12-0311-82	1:428	FACS
Monoclonal mouse-anti-CD45	eBIOSCIENCE #12-0451-83	1:428	FACS
Monoclonal mouse-anti-Sca1	eBIOSCIENCE #12-5981-82	1:428	FACS
Monoclonal mouse-anti-TER-119	eBIOSCIENCE #12-5921-82	1:428	FACS
Polyclonal rabbit anti-total TAK1	Cell Signaling Technology # 4505	1:1000	WB
Polyclonal rabbit anti-total TAB1	Cell Signaling Technology # 3225	1:1000	WB
Polyclonal rabbit anti-phospho-ERK1/2	Cell Signaling Technology # 9101	1:1000	WB
Polyclonal rabbit anti-total-ERK1/2	Cell Signaling Technology # 9102	1:1000	WB
Polyclonal rabbit anti-phospho-JNK1/2	Cell Signaling Technology # 9251	1:1000	WB
Polyclonal rabbit anti-total-JNK1/2	Cell Signaling Technology # 9252	1:1000	WB
Polyclonal rabbit anti-phospho-p38	Cell Signaling Technology # 9211	1:500	WB
Polyclonal rabbit anti-total p38	Cell Signaling Technology # 9212	1:1000	WB
Monoclonal rabbit-anti-phospho-IκBα	Cell Signaling Technology # 2859	1:500	WB
Monoclonal rabbit-anti-total IκBα	Cell Signaling Technology # 4812	1:1000	WB
Monoclonal-rabbit-anti-cleaved caspase 3	Cell Signaling Technology # 9664	1:1000	WB
Polyclonal rabbit-anti-cleaved PARP	Cell Signaling Technology # 9544	1:1000	WB
Polyclonal rabbit-anti-p100/p52	Cell Signaling Technology # 4882	1:500	WB
Monoclonal rabbit-anti-GAPDH	Cell Signaling Technology # 2118	1:2000	WB
Monoclonal rat-anti-MLKL	Millipore, MABC604	1:1000	WB
Polyclonal goat-anti-FADD	Santa Cruz Biotechnology, sc-6035	1:500	WB
Polyclonal rabbit-anti-c-IAP1	Santa Cruz Biotechnology, sc-7943	1:500	WB
Monoclonal mouse-anti-Bcl-2	BD Biosciences #556358	1:1000	WB
Polyclonal rabbit-anti-Cyclin D3	Santa Cruz Biotechnology, sc-182	1:500	WB
Monoclonal mouse-anti-Cdc42	Santa Cruz Biotechnology, sc-8401	1:500	WB
Monoclonal mouse-anti-Pax7	DSHB Pax7	1:1000	WB
Monoclonal mouse-anti-eMyHC	DSHB F1.652	1:1000	WB
Polyclonal rabbit-anti-MyoD	Santa Cruz Biotechnology, sc-304	1:1000	WB
Polyclonal rabbit-anti-myogenin	Santa Cruz Biotechnology, sc-576	1:500	WB
Monoclonal mouse-anti-RIP	BD Biosciences #610458	1:500	WB
Monoclonal mouse-anti-eMyHC	DSHB F1.652	1:250	Immunohistochemistry
Polyclonal rabbit-anti-Laminin	Sigma, L9393	1:500	Immunohistochemistry
Monoclonal mouse-anti-Pax7	DSHB Pax7	1:10	Immunohisto/cyto-chemistry
Polyclonal rabbit-anti-MyoD	Santa Cruz Biotechnology, sc-304	1:200	Immunocytochemistry
Monoclonal rat-anti-TNF-α	GeneTex, MAB0856	1:20	Neutralization

Supplementary Table 2. Sequence of the primers used for QRT-PCR analyses.

**Gene**	**Sequence (5' → 3')**
TAK1	GTCATCCAGCCCTAGTGTCAGAAT (Forward)<br>TTCTTTGGAGTTTGGGCACG (Reverse)
TNFα	GCATGATCCGCGACGTGGAA (Forward)<br>AGATCCATGCCGTTGGCCAG (Reverse)
TNFRI	AACCAGTTCCAACGCTACCTGA (forward)<br>AGAAAGAACCCTGCATGGCA (reverse)
TNFRII	TAAGTGCCATCCCAAGGACACTCT (forward)<br>CCCAGTGATGTCACTCCAACAATC (reverse)
TWEAK	GCTACGACCGCCAGATTGGG (forward)<br>GCCAGCACACCGTTCACCAG (reverse)
IL-6	CCTTCTTGGGACTGATGCTGG (forward)<br>GCCTCCGACTTGTGAAGTGGT (reverse)
Pax-7	CAGTGTGCCATCTACCCATGCTTA (Forward)<br>GGTGCTTGGTTCAAATTGAGCC (Reverse)
Myf5	TGAAGGATGGACATGACGGACG (Forward)<br>TTGTGTGCTCCGAAGGCTGCTA (Reverse)
MyoD	TGGGATATGGAGCTTCTATCGC (Forward)<br>GGTGAGTCGAAACACGGATCAT (Reverse)
Myogenin	CATCCAGTACATTGAGCGCCTA (Forward)<br>GAGCAAATGATCTCCTGGGTTG (Reverse)
Myh3	ACATCTCTATGCCACCTTCGCTAC (Forward)<br>GGGTCTTGGTTTCGTTGGGTAT (Reverse)
HeyL	CAGATGCAAGCCCGGAAGAA (Forward)<br>ACCAGAGGCATGGAGCATCT (Reverse)
Hes1	GCACAGAAAGTCATCAAAGCC (Forward)<br>TTGATCTGGGTCATGCAGTTG (Reverse)
Hes6	GCCGGATTTGGTGTCTACAT (Forward)<br>TCCTGAGCTGTCTCCACCTT (Reverse)
β-actin	CAGGCATTGCTGACAGGATG-3' (Forward)<br>TGCTGATCCACATCTGCTGG (Reverse)
